# On the formation of superparamagnetic iron oxide nanoclusters

**DOI:** 10.1039/d6ra04862e

**Published:** 2026-10-07

**Authors:** Philipp Schwarz, Heinz Amenitsch, Joachim Bansmann, Johannes Biskupek, Mika Lindén

**Affiliations:** a Institute of Inorganic Chemistry II, Ulm University Albert-Einstein-Allee 11 89081 Ulm Germany mlinden@uni-ulm.de; b Institute for Inorganic Chemistry, Graz University Stremayrgasse 9/IV 8010 Graz Austria; c Institute of Physical Chemistry II, Ulm University Albert-Einstein-Allee 47 89081 Ulm Germany; d Central Facility Electron Microscopy, Ulm University Albert-Einstein-Allee 11 89081 Ulm Germany

## Abstract

Even though clusters consisting of superparamagnetic iron oxide nanoparticles (SPION's) have been widely studied, means for rational tuning of important parameters such as crystallite size and aggregate packing density are rarely discussed. Both the crystallite size and the nanocluster size are important parameters for many applications involving SPION's, and the presented synthesis strategy allows for optimization of the SPION nanoclusters for a given application. Here we show that these parameters can be partially independently tuned through controlled water addition to a synthesis of cationically stabilized, monodisperse nanoclusters of SPION's in the presence of polyethylene imine (PEI). Larger crystallites form with increasing water content, which increases the contribution of attractive van der Waals interactions. The crystallize size within already formed nanoclusters can be further increased through increasing the synthesis time. On the other hand, larger nanoclusters with comparable crystallite sizes form in the absence of PEI. Based on DLS, XRD, TEM and SAXS measurements of the synthesized nanoclusters, a mechanism of formation is proposed that highlight the importance of the crystallite size of the primary particles before cluster formation as a key parameter for achieving well-defined, spherical nanoclusters, and that the critical crystallite size is dependent on the magnitude of electrostatic repulsion for a given system.

## Abstract 2

Even though clusters consisting of superparamagnetic iron oxide nanoparticles (SPION's) have been widely studied, means for rational tuning of important parameters such as crystallite size and aggregate packing density are rarely discussed.

## Graphical abstract

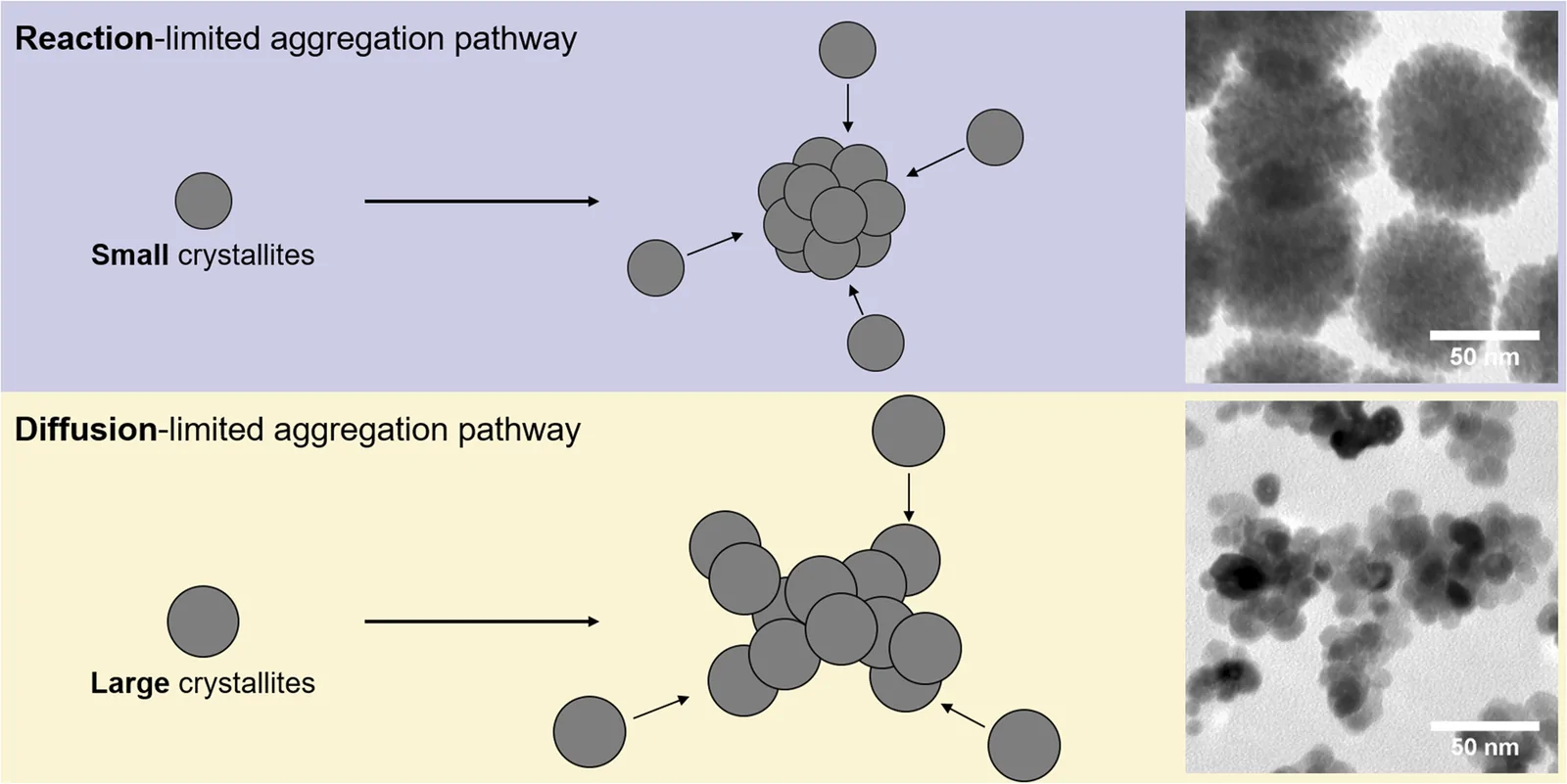

## 1. Introduction

Monodisperse aggregates of several hundred to thousands of superparamagnetic iron oxide nanocrystallites, often also called nanoclusters,^1,2^ multicore particles,^3,4^ nanoflowers^5–7^ or nanocrystal clusters,^8^ have gained attention as the nanoconstruct of choice when it comes to bioapplications of iron oxide nanoparticles.^9–11^ These nanoclusters are typically solvothermally synthesized in the presence of polymer, and form due to balancing short-range attractive dispersion forces and long-range repulsive electrostatic forces.^12–21^ These nanoclusters have total dimensions exceeding the superparamagnetic size limit, but simultaneously alter the magnetic properties compared to single crystallite particles due to cooperative effects of the crystallites within the cluster.^1,6,8,22–26^

The cluster size as well as crystallite size are important parameters for optimization, as the crystallite size or even the crystallite size distribution can greatly affect relaxation properties in MRI,^27^ the openness of the cluster influences its contact area with the solvent^28^ as well as its adsorption properties, and the cluster size influences the biodistribution of the clusters.^29–35^ Various studies also demonstrate that nanocluster size, crystallite size and number of crystallites per nanocluster (*i.e.* crystallite packing density) can be important parameters for hyperthermia applications as well.^4,6,7,36,37^ Despite the large number of synthesis protocols that have been published leading to the formation of nanoclusters with different morphologies, a holistic mechanistic framework describing rational means for parallel tuning of crystallite size, cluster porosity/surface area, and cluster sizes is still missing. However, the crystallite size seems to scale with both the water content in the synthesis procedure^38–42^ as well as the reaction time.^5,43–45^ This has been ascribed to a faster hydrolysis of the precursor in the presence of water, and to continuous growth of the crystallites through ripening. An increase in reaction temperature serves the same purpose.^46^ However, the factors that control cluster size are less clear.^9^ Several studies observe a decreased nanocluster size upon addition of water to the synthesis mixture, and ascribe this to an increase in the number of reaction/nucleation centers.^42,46,47^ Others suggest the concentration of the surface molecules might be decisive in order to balance the aggregation process.^48^ With a higher citrate concentration for example, smaller nanoclusters are created due to higher repulsion between the crystallites.^12,49,50^ It should be noted that this higher surface molecule coordination is often accompanied by a decrease in crystallite size as well, making the interpretation of the resulting effect more difficult. Similar results can be achieved with an increase of the iron precursor concentration, resulting in a lower surface-molecule-to-iron ratio or more nucleation sites.^49,51,52^ Another parameter that is often utilized is the solvent. Most iron oxide nanocluster synthesis approaches include glycol solvents. By changing the glycol species or the ratio between different glycols, a significant change in nanocluster size can be observed. For example, a partial replacement of ethylene glycol with diethylene glycol is often used to create smaller nanoclusters.^41,50,53–56^ These observations are usually ascribed to the differences in viscosity when the glycol mixture is altered.^41,50,54,57^ Even though the nanocluster size changes are evident, it is questionable whether viscosity can explain these findings, since the differences in viscosity between the glycols are very low at elevated temperatures.^58^

Here we show that the crystallite and nanocluster size of cationic iron oxide nanoclusters can be independently tuned to some extent through variations of the water content reaction mixture. The resulting positively charged nanoparticles are interesting candidates in their own right for use in (magnetic) transfection^59–64^ and gene delivery in general,^65,66^ and are suitable for further adsorptive or covalent biofunctionalization.^67–70^ Next to that, due to the independent size modulation, we can investigate the influence of the crystallite size as well as the polyethylene imine (PEI) on the aggregation into nanoclusters through a series of synthesis procedures with varying conditions. Through this approach, we hypothesize that we can tune the morphology and derive implications revolving around the formation mechanism. Especially the crystallite size as a parameter in the controlled aggregation is highlighted.

## 2. Experimental section

### 2.1 Chemicals

Iron(iii) acetylacetonate (Fe(acac)_3_, 99%) and diethylene glycol (DEG, 99%) were purchased from Thermo Fisher. Diethylene glycol (DEG, 99%), triethanol amine (TEA, 99%) and branched polyethylene imine (PEI, MW 600–800) were purchased from Sigma-Aldrich. All chemicals were used without further purification.

### 2.2 Synthesis of iron oxide nanoclusters

Iron oxide nanoclusters were synthesized according to a modified literature procedure.^53,71^ 2 g (57 mmol) Fe(acac)_3_ was dissolved in 100 mL DEG in a 250 mL round-bottom flask. 0.5–2.0 mL MilliQ water was added to adjust the water amount in the mixture before heating to 80 °C over the course of 30 min. Under constant stirring. 1.5 g (2 mmol) PEI was added, and the mixture was homogenized for 40 min at 80 °C under stirring, if not mentioned otherwise. Finally, 6.74 g (45 mmol) TEA was added, and the reaction mixture was stirred for another 40 min. The mixture was then transferred to a 200 mL Teflon vessel, sealed in a steel autoclave, and heated at 200 °C for 12 h (heat ramp 3 K min^−1^) under static conditions. The resulting particles were magnetically separated and washed three times with demineralized water and twice with isopropanol. The particles were subsequently dried in air at 50 °C for 1–2 h.

### 2.3 Characterization

DLS and zeta potential measurements were done with a Malvern Zetasizer Nano-ZS ZEN 3600. The samples were dispersed 5 min in a Covaris S220 focused ultrasonication bath beforehand. For DLS, particle dispersions of 0.01 mg mL^−1^ in demineralized water were prepared and measured at 633 nm wavelength and a backscattering angle of 173°. For every sample, 3 rounds of 10 single measurements at 25 °C were done. If not mentioned otherwise, the number weighted mean value of the 30 measurements along with the standard deviation of this mean value is used in the results section. Zeta-potential measurements were performed at a concentration of 0.1 mg mL^−1^ in an aqueous 1 mM KCl solution. The reported value corresponds to the mean value of 13 single measurements together with the measured zeta deviation. Transmission electron microscopy (TEM) images were recorded at a JEOL JEM-1400 at 120 kV. Samples were prepared as a dispersion in isopropanol, then one drop is placed onto a copper grid and dried at room temperature. For high resolution TEM (HRTEM), the samples were dispersed in ethanol, super-sonicated, and subsequently drop-casted on holey carbon film support grids. HRTEM and scanning (S) TEM investigations were carried out using a Thermo Fisher Talos 200X microscope at 200 kV accelerating voltage. HRTEM images were acquired using a CETA 2 CMOS camera. High angle annular dark field (HAADF) STEM images were acquired together with the parallel acquisition of energy dispersive X-ray (EDX) spectra (Thermo Fisher SuperX EDX detector). The Velox software package was used the create local elemental maps and to quantify the stoichiometry. Nitrogen sorption measurements were performed using a Quantachrome Quadrasorb-SI setup. Samples were heated for 24 h at 60 °C under vacuum prior to the measurement. Quantification of the surface area was done with BET method. XPS analysis was performed using a commercial XPS (PHI 5800 from Physical Electronics). It consists of a hemispherical condenser and a monochromatized Al Kα radiation source. The apparatus is additionally equipped with an Ar ion sputter gun for depth profiling and a neutralizer to compensate for charging in case of samples with insufficient conductivity. The spectra were recorded with a path energy of 93.9 eV (survey spectra) and 58.7 eV in case of detailed spectra. Raman spectra were recorded with a WITec alpha300 R using a YAG-laser (WITec, 532 nm, 0.1 mW) and a R-32413 laser (REO, He–Ne, 633 nm, 0.5 mW). Magnetization curves were recorded with a Lakeshore VSM 7310 at 300 K with magnetic fields of −15000 to 15 000 G. After background subtraction, magnetization values were normalized to the pure iron oxide mass derived by thermogravimetry measurements. XRD measurements were done at a PANalytical X'Pert PRO with Cu Kα (154 pm) with 0.01° steps and 30 s count time per step. Instrumental peak broadening was determined by measuring a silicon standard. The crystallite size was calculated either through the Scherrer equation or a size-strain plot analysis. In case of the Scherrer method and the size-strain analyses, a shape factor value of 0.9 was assumed. The FWHM and *θ* was derived through a Gauss-fit of the (311) reflection. The size-strain plot analysis was done through the following equation according to literature:^72^1
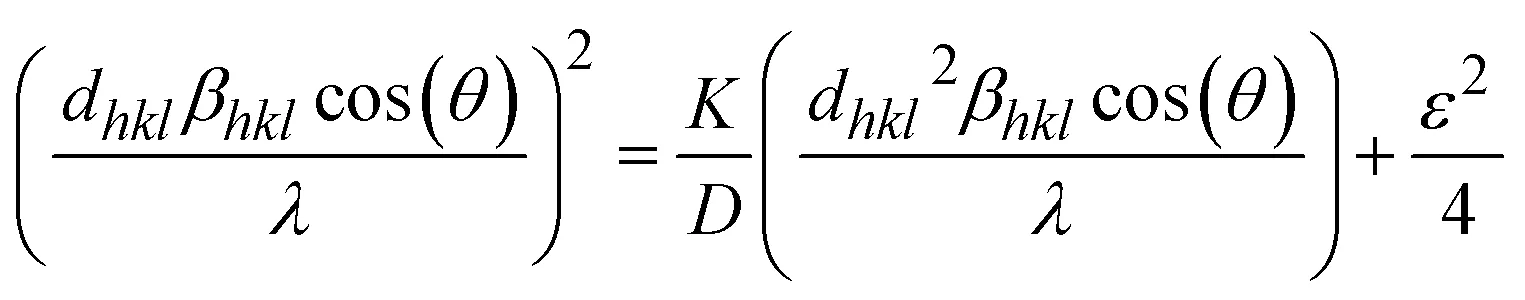
*d*_*hkl*_ represents the *d*-spacing of the reflection, *β*_*hkl*_ the respective full-width-half-maximum based on a pseudo-Voigt regression corrected for instrumental broadening and cos(*θ*) the position. *K* is assumed to be 0.9. The wavelength *λ* is 0.154 nm. The crystallite size *D* and strain *ε* are approximated based on the slope and *y*-intercept of the linear regression based on the most intense reflections (220, 311, 400, 422, 511 and 440). The specific surface area (*A*_S_) in m^2^ g^−1^ was calculated by the area (*A*) divided by the mass (*m*) of the sample. The maximum possible geometric specific surface area was then be determined based on the crystallite size or rather the radius (*r*_XRD_ = *D*/2) determined by XRD and approximating the crystallites as spheres with a density *ρ* of 5.0 g cm^−3^ using the equation:2
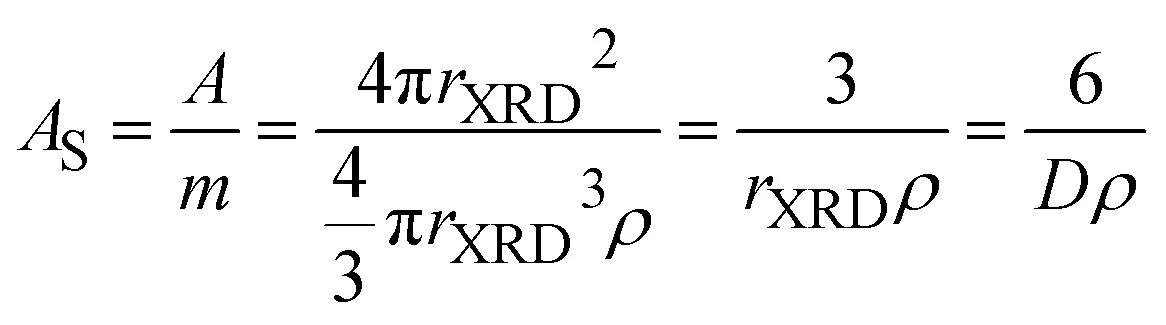


SAXS measurements were performed on a Bruker AXS Nanostar equipped with a Vantec-2000 detector using Cu Kα radiation. The data was normalized and transmission corrected with standard procedures based on reference measurements with glassy carbon. The SAXS data was analyzed using a simplified hierarchical structural model consisting of large spherical structures composed of smaller spherical particles (crystallites), which have been analyzed separately. In the SAXS scattering analysis of both structural levels, polydispersity was accounted for by assuming a Gaussian size distribution of the radii (radius *R*_x_, relative width of the size distribution *σ*_rx_ = polydispersity index PDI = *σ*_x_/*R*_x_ as volume size distribution) defined by3
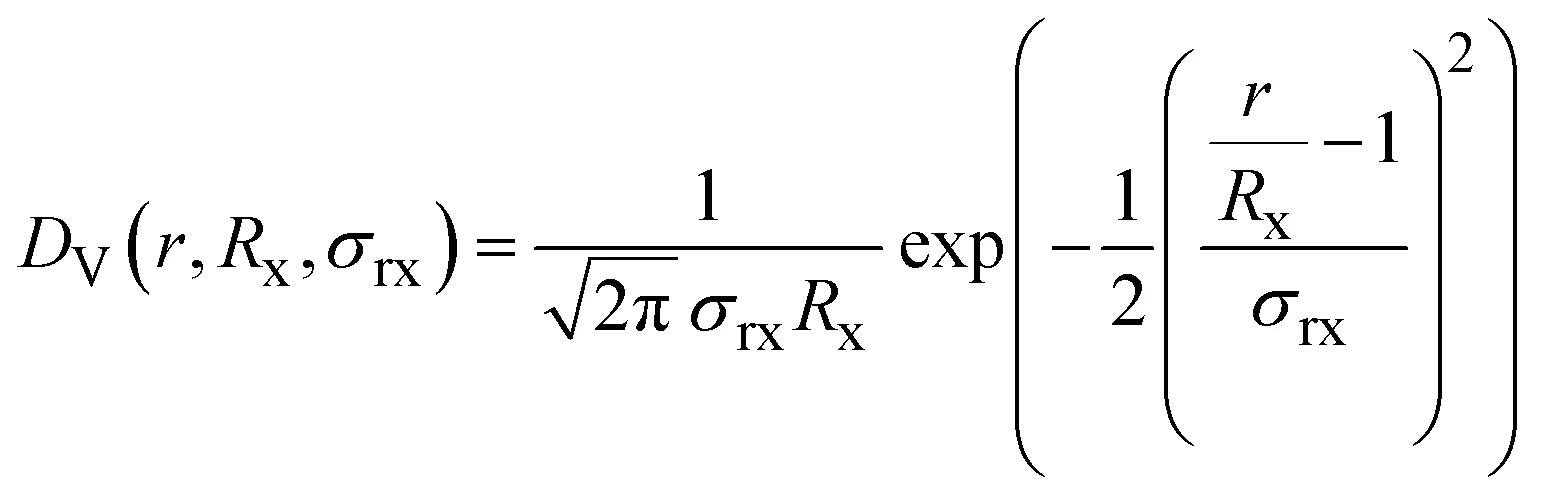


To describe correlations between the small particles, fractal structure factors were introduced to capture the hierarchical structural arrangement, ranging from more compact (surface fractal like) to more open configurations (mass fractal like). The structure factor for mass fractals *S*_M fract_(*q*, *R*_0_, *ξ*, *D*_v_),^73^ has the form as given in the eqn (4). The parameters *R*_0_, *ξ*, and *D*_v_ represent the radius of the structural building units (*i.e.*, the small crystallites, *R*_c_), the correlation length associated with the cut-off distance, and the fractal dimension *D*_v_ corresponding to the power-law exponent *α*, respectively. The structure factor for surface fractals *S*_S fract_(*q*, *A*, *D*_s_) with *A* as a scaling factor, and *D*_s_ the dimension of the surface fractal corresponding to the power-law exponent *α* = 6 − *D*_s_ is given in eqn (5). The approach of adding the auto correlation contribution to the structure factor for surface fractals as in the work of Bale and Schmidt^74^ was following the argumentation as described for mass fractals.^73^4
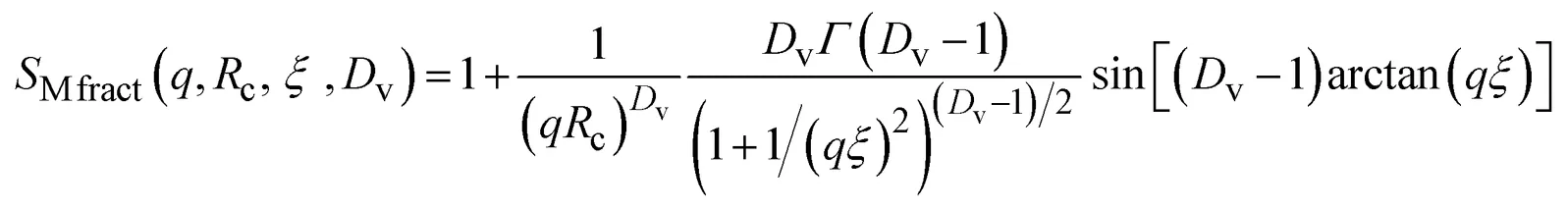

5 *S*_S fract_(*q*, *A*, *D*_s_) = 1 + *AΓ*(5 − *D*_s_) sin[π(*D*_s_ − 1)/2]/*q*^6−*D*_s_^

The form factor *P*(*q*, *R*_x_, *σ*_rx_) is given by eqn (6), assuming spherical particles with the Gaussian volume size distribution *D*_v_(*r*) as defined in eqn (3).^75^ The scattering function for the small crystallites *I*_sas S_(*q*) is given by the product of the form factor and the corresponding fractal structure factor (surface or mass fractal),^76^ as described by:6
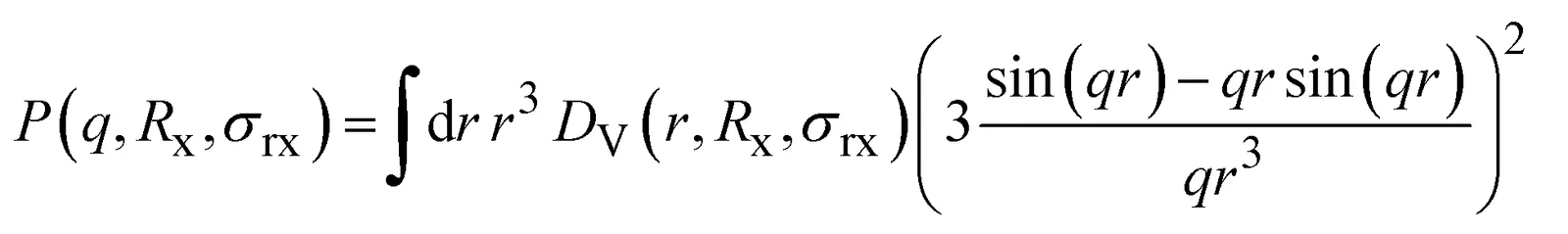

7 *I*_sas S_(*q*) = *I*_0c_*S*_X fract_(*q*, Par_j_)*P*(*q*, *R*_c_, *σ*_c_) + bkg_S_

where *I*_0c_ is the forward scattering intensity of the crystallites, Par_j_ are the parameters of the corresponding structure factor, *R*c is their mean size and *σ*_*r*c_ the relative width of the gaussian volume size distribution. A constant background bkg_S_ is added to account for residual background scattering at high *q-*values.

The size of the aggregate structure was determined using either of two approaches: (i) by fitting the *S*_M fract_(*q*, *R*_c_, *ξ*, *D*_v_) with the form-factor contribution from the crystallites over the entire *q*-regime and interpreting the correlation length *ξ* of the mass fractal as size parameter for the large aggregates; or (ii) by fitting the first side minima of the volume-size-distribution-smeared-spherical particle-scattering function to the scattering data (around *q* = 0.15 nm^−1^) following the approach of eqn (6).

In the latter case, the fit also included a background contribution consisting of a power law term and a constant background term representing an empirical contribution from the small crystallites in the *q*-range 0.118 to an upper limit between 0.25–0.6 nm^−1^ depending on the *q*-range over which the power law scattering from the large aggregates remained significant. The corresponding expression is:

8 *I*_sas L_(*q*) = *I*_0A_*P*(*q*, *R*_A_, *σ*_rA_) + *c*_p_/*q*^pe^ + bkg_L_

with *I*_0A_ the forward scattering of the large aggregates, *R*_A_ the mean radius and *σ*_rA_ the relative relative width of the Gaussian volume size distribution of the large aggregates. The effective contribution of the small crystallites is given by *c*_p_ and *p*_e_ power-law constant and exponent, as well as bkg_L_ as constant background.

## 3. Results

### 3.1 Influence of water addition

In the absence of added water, the synthesis yields monodisperse nanoclusters with a mean diameter of about 80 nm according to TEM and DLS (Fig. 1a and S1a). The surface of these nanoclusters is functionalized with PEI (Fig. S1b and S4) while XRD analysis suggest that the inorganic material is are either made of magnetite (Fe_3_O_4_, PDF-Nr. 98-008-4611),^77^ maghemite (γ-Fe_2_O_3_, PDF-Nr. 98-024-7036)^78^ or of a mixture of both (Fig. 1f). XPS analysis indicates the presence of mostly magnetite, due to the presence of Fe(ii), while Raman spectroscopy analysis performed using two different excitation wavelengths confirmed the presence of both maghemite and magnetite signals (Fig. S2 and S3). This would be in agreement with literature studies showing that iron oxide nanoparticles tend to form an oxidized outer layer.^79–84^ Vibrating sample magnetometer (VSM) measurements confirmed that the nanoclusters were superparamagnetic, with a high saturation magnetization of 70 emu g^−1^ combined with a low coercivity and remanence (Fig. S5).

**Fig. 1:**
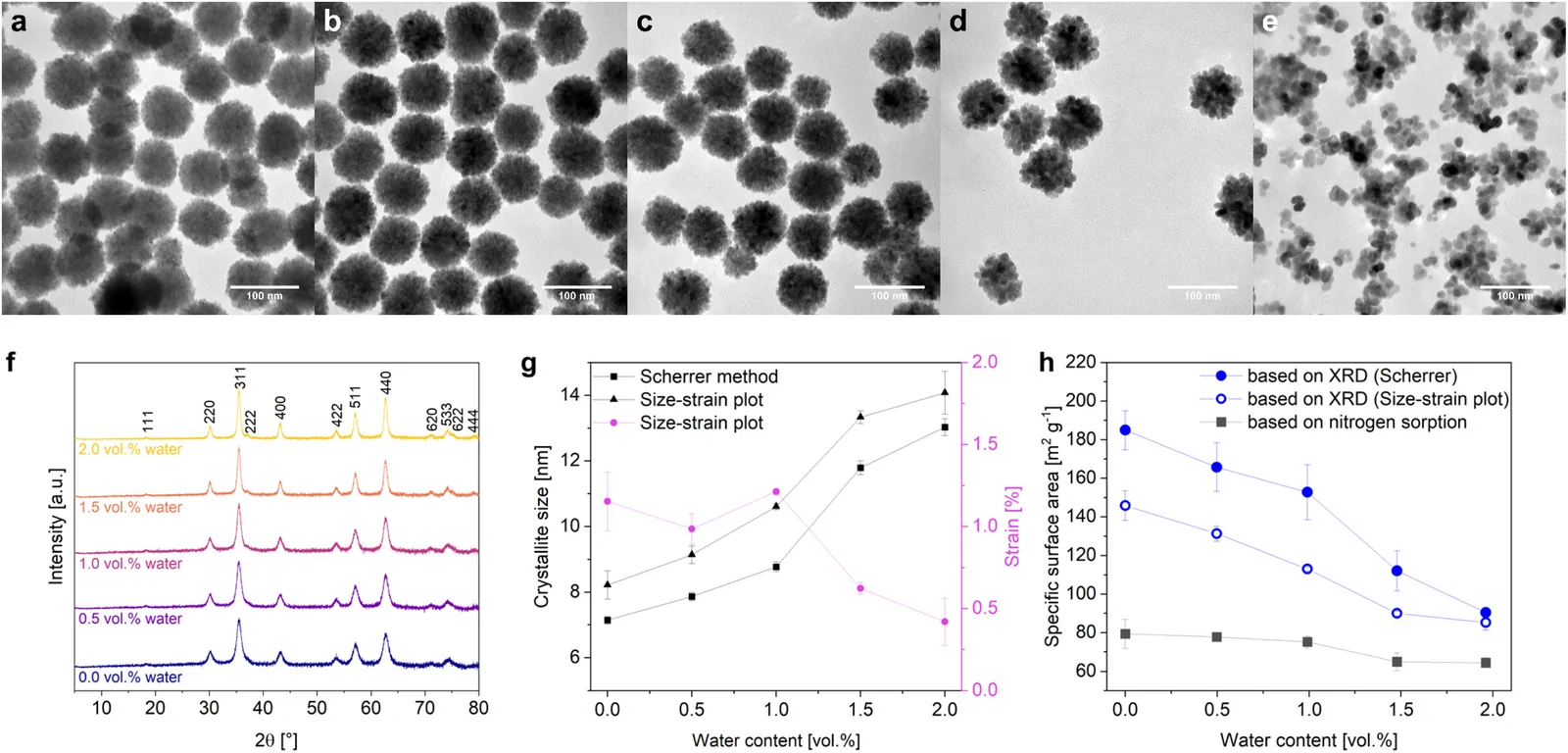
TEM images of the nanoclusters synthesized without (a), 0.5 vol% (b), 1.0 vol% (c), 1.5 vol% (d) and 2.0 vol% (e) water. Based on the XRD, the iron oxide composition (f) remains comparable throughout the different synthesis conditions along with a peak narrowing indicating growing crystallites. This narrowing can be either attributed to only the crystallite size in case of the Scherrer method or differentiated into a size and strain effect through a size-strain plot (g). The difference between the calculated specific surface area of the nanoclusters is compared to the measured surface area by nitrogen sorption changes, indicating a change in the accessible surface area through water addition during synthesis (h).

If water is added to the sol, an increased surface roughness of the clusters (Fig. 1a–e) was observed, while the overall nanocluster size remained relatively constant in the range 75–85 nm up to a total water amount of 1.5 vol% (Fig. 1a–d and S1a), without any additional structural or compositional changes. This was accompanied by growth of the mean primary crystallite sizes inside the nanoclusters from 7–8 nm up to 12–13 nm (Fig. 1f and g) as determined by Scherrer or size-strain analyses (Table S1 and Fig. S6 (Williamson–Hall) and S7 (SSP) analyses). At a water content of 2.0 vol% only poorly morphologically defined nanoclusters with mean primary crystallite sizes in the range of 13–14 nm are formed (Fig. 1e). The change in morphology was also seen in the mean hydrodynamic diameters determined by DLS, where a constant value of about 75 nm was obtained up to a water content of 1.5 vol%, which decreased to about 30 nm for particles synthesized in the presence of 2.0 vol% water (Fig. S1a).

The specific surface area of the different samples was determined by nitrogen sorption (Fig. 1h). With increasing water addition from 0.0 vol% to 2.0 vol%, the surface area decreased from 79 m^2^ g^−1^ to 64 m^2^ g^−1^. Also included are values calculated based on the crystallite size determined by XRD under the assumption that the surface of the crystallites assumed to be monodisperse is fully accessible, and that the crystallites are spherical and exhibit a density of 5.0 g cm^−3^. Thus, this value can be seen as the maximum theoretical surface area. This maximum surface area then depends on whether the XRD size values from the Scherrer method or the size-strain analysis are used. Nevertheless, the so calculated surface area values are significantly higher than the BET areas for all samples. In absence of additional water, the nanoclusters are tightly packed as can be seen through the large discrepancy of nitrogen sorption results and calculated surface areas. But the difference becomes smaller with increasing water content, and especially so for the 2.0 vol% water sample, again suggesting that the presence of water leads to the formation of more open structures with a higher accessible surface, as suggested by the TEM images.

High-resolution TEM imaging confirmed that the primary particles of the nanoclusters are single crystallites (Fig. 2 and S9). In the absence of added water, the crystallite sizes were 6–7 nm and increased with addition of water to about 9–12 nm (1.5 vol%) and about 11–20 nm (2.0 vol%), respectively (Fig. S10a). These values are in good agreement with those derived based on XRD analyses and additionally suggest that the crystallite size distribution broadens with increasing water content. Furthermore, Fast Fourier Transform (FFT) analyses (Fig. 2d–f and S9) indicated that the crystallites in the clusters synthesized without water addition exhibited a high degree of local alignment based on the appearance of only few broad signals. The broadening however still suggested some degree of twisting between neighbouring crystallites. This would be indicative for cluster growth based on oriented attachment of the single crystallites. With 1.5 vol% and 2.0 vol% water content in the synthesis, the orientation of the crystallites was rather random, indicated by an increasing number of narrow spots in the FFT. This then would correspond to a random aggregation of the crystallites during nanocluster formation.

**Fig. 2:**
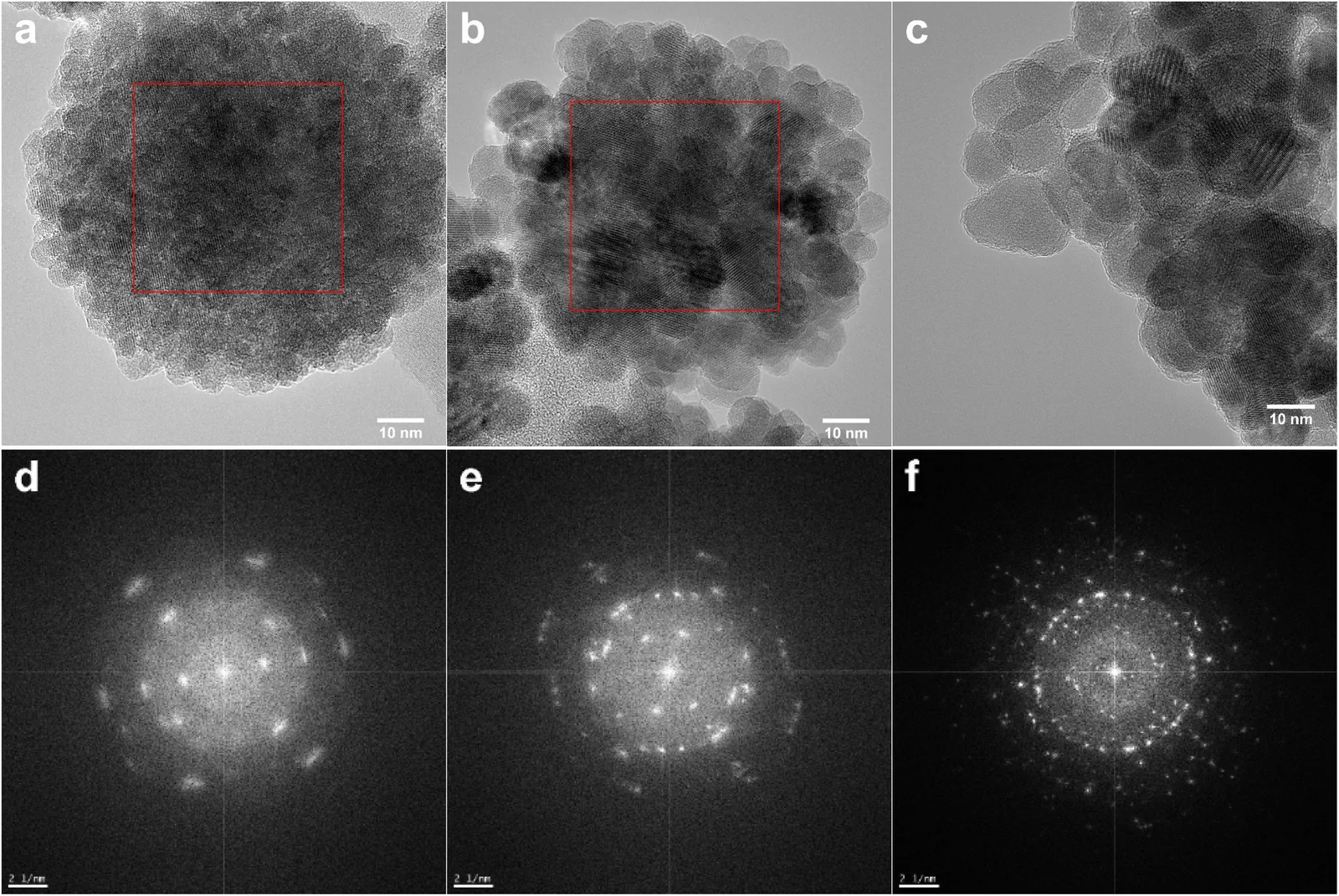
HRTEM images of the nanoclusters synthesized at 0.0 vol% water (a), 1.5 vol% water (b) and 2.0 vol% water (c) along with arrows indicating the growing size of the crystallites. Local fast Fourier transformation images of the respective nanoclusters show that with increasing water content in the reaction mixture, the orientation between the crystallites inside one nanocluster shifts from a rather uniform distribution in absence of additional water (d) to a more random distribution with 1.5 vol% (e) and 2.0 vol% water added (f). The areas used for the FFT are indicated. For (f), the whole image was used for the FFT calculation.

We further carried out SAXS measurements to gain a deeper insight into the hierarchical architecture of the crystallites and clusters (Fig. 3). Also included are fits to the data based on a hierarchical two-particle size model approximated with a fractal structure factor of the small particles. The details of the structure determination of the clusters and crystallites are outlined in the experimental section and in the SI (Fig. S11). Key-parameters derived from the fits and the complementary measurements (XRD, TEM, DLS, SAXS) are summarized in Table 1. Although the crystallite sizes derived from different techniques differ, the general trend in size increase remains in all measurement techniques. Differences between mean crystallite size values determined by TEM and XRD can be understood, as TEM delivers number-weighted values while XRD delivers volume-weighted values. Furthermore, oriented attachment of adjacent crystallites can lead to an increase in the crystallite sizes determined by XRD.^23,83^ The latter contribution is the highest for the samples synthesized at low water contents, and it is also for these samples that the crystallite size values differ the most between values derived by XRD and TEM. On this note, the agreement between the crystallite sizes derived from the SAXS analysis and TEM is much better, further supporting the suggestion that oriented attachment effects lead to an overestimation of the XRD-derived crystallite sizes at low water content. The power law coefficients of −3.8 to −4.0 associated with the cluster structures for water contents up to 1.5 vol%, analysed with a surface fractal fit, were characteristic for surface fractal structures expected for monomer-cluster growth, in good agreement with the TEM images. For the 2.0 vol% water sample, analysed with a volume fractal fit, a clearly lower power law coefficient of about −2.4 was obtained, characteristic of an open mass fractal structure, again in good agreement with the TEM data.^85,86^

**Fig. 3:**
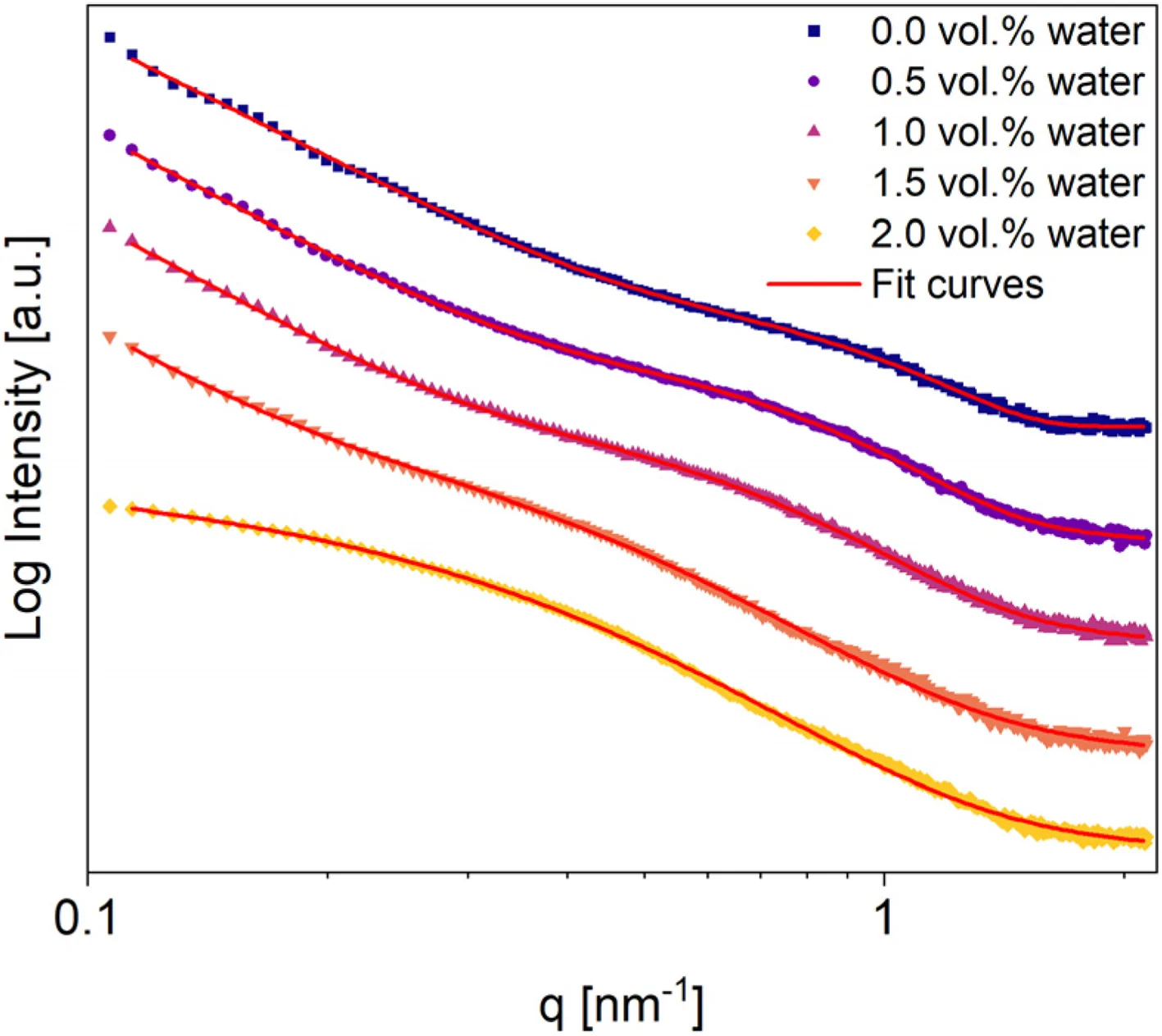
The SAXS curves of the various nanoclusters represent a superposition of the scattering curves of the smaller crystallites and the larger nanocluster structures approximated by fractal structure factors (shown as curve fits – eqn (7)).

**Table 1:** Comparison of different parameters derived by several measurement techniques

Water added [vol%]	Crystallite analysis					Cluster analysis					
	Size (XRD – Scherrer method) [nm]	Size (XRD – size-strain plot) [nm]	Strain (XRD – size-strain plot) [%]	Size (HRTEM; *n* = 50) [nm]	Size (SAXS) [nm]	Size (DLS – number) [nm]	PDI (DLS) [nm]	Size (TEM; *n* = 200) [nm]	Size standard deviation (TEM; *n* = 200) [nm]	Size (SAXS) [nm]	Power law (SAXS)
0.0	7.1	8.2	1.2	5.6	5.0	86	0.06	85	12	79	−3.8
0.5	7.8	9.1	1.0	—	5.8	81	0.08	89	12	88	−4.0
1.0	8.8	10.6	1.2	—	6.6	79	0.08	84	12	79	−4.0
1.5	11.7	13.3	0.6	10.0	10.4	76	0.08	79	13	67	−4.0
2.0	12.9	14.1	0.4	15.0	11.4	36	0.21	—	—	45	−2.4

### 3.2 Synthesis without PEI

As adsorbed PEI contributes to the electrostatic repulsion interactions which should oppose nanocluster growth, syntheses were also carried out in the absence of PEI as a function of water content. Well-defined clusters were obtained up to a water content of 1.0 vol% (Fig. 4a and b), but with an increased mean nanocluster size as compared to corresponding syntheses carried out in the presence of PEI, as also confirmed by DLS (Fig. 4d). In the absence of added water, the mean nanocluster size was now around 100 nm (compared to 80 nm in the presence of PEI), and the size increased up to 160 nm at a water content of 1.0 vol%, while it remained constant at 80 nm in the presence of PEI. Furthermore, the surface roughness increased with increasing water content, as also seen for the nanoclusters synthesized in the presence of PEI. At a water content of 1.5 vol% only smaller, irregular clusters were observed, as in the case of the PEI-containing syntheses (Fig. 4c) carried out at a water content of 2.0 vol%. The formation of irregular clusters was also seen in the DLS measurements, which exhibited a clearly smaller hydrodynamic diameter than the clusters synthesized at lower water contents (Fig. 4d). The mean crystallite sizes increased slightly from 6.8 nm with increasing water content up to 9.3 nm at 1.0 vol% water, while a clearly larger crystallite size of 11.0 nm was seen for the irregular aggregates based on size determination with the Scherrer method. Meanwhile, the size-strain plot analysis indicates a size increase from 9.0 nm up to 10.4 nm with 1.0 vol% and eventually 12.0 nm with 1.5 vol% added water (Fig. S7c). In all cases, XRD analyses showed the sole presence of maghemite/magnetite (Fig. 4e).

**Fig. 4:**
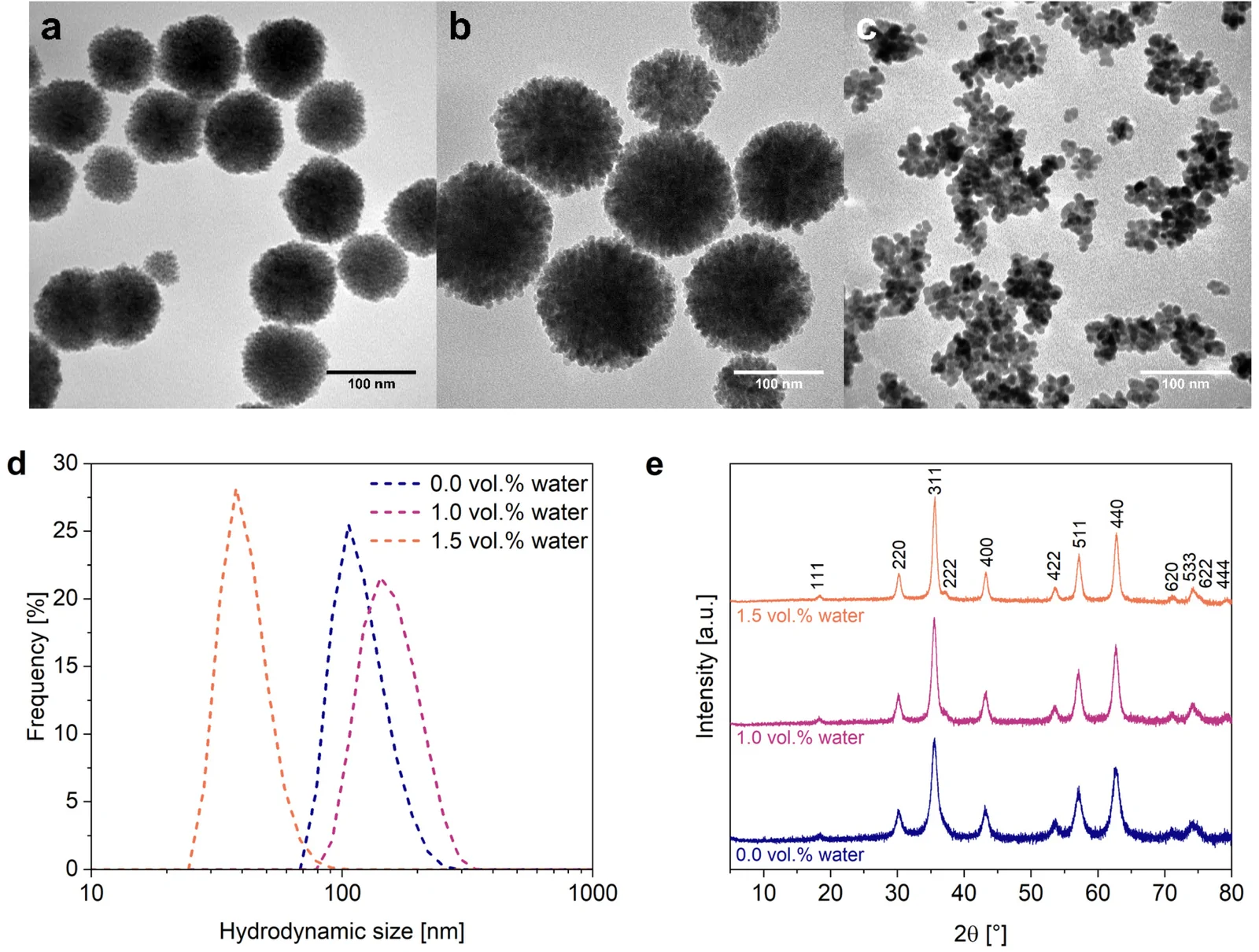
(a)–(c) TEM images showing the morphology of the nanoclusters. Comparison of the nanoclusters synthesized in the absence of PEI (a) and with additional water of 1.0 vol% (b) and 1.5 vol% (c). When water is added, the overall nanocluster size increases at first but then leads to irregular structures at higher water contents (d). The crystallite composition remains comparable in absence of PEI and with increasing water content (e).

### 3.3 Influence of reaction time

The influence of reaction time was evaluated for PEI-containing syntheses up to a water content of 1.5 vol% (Fig. 5a–i), *i.e.* for syntheses leading to well-defined spherical aggregates. Prolonging the reaction time led to an increase in crystallite size but had little influence on the cluster size (Fig. 5j, S8 and S10b). This was especially true if water is present in the reaction mixture, indicating that the presence of water enhances the ripening kinetics. In the synthesis without additional water, the crystallites grow with around 0.1 nm h^−1^. At 1.0 vol% water, this increases to 0.3 nm h^−1^ and reaches 0.4 nm h^−1^ at 1.5 vol% water added (Fig. 5k). The constant cluster size despite differences in crystallize size suggests that crystal growth occurs through ripening within already formed nanoclusters during aging. This sort of “intracluster” – ripening has also previously been described in the literature.^5,43–45^

**Fig. 5:**
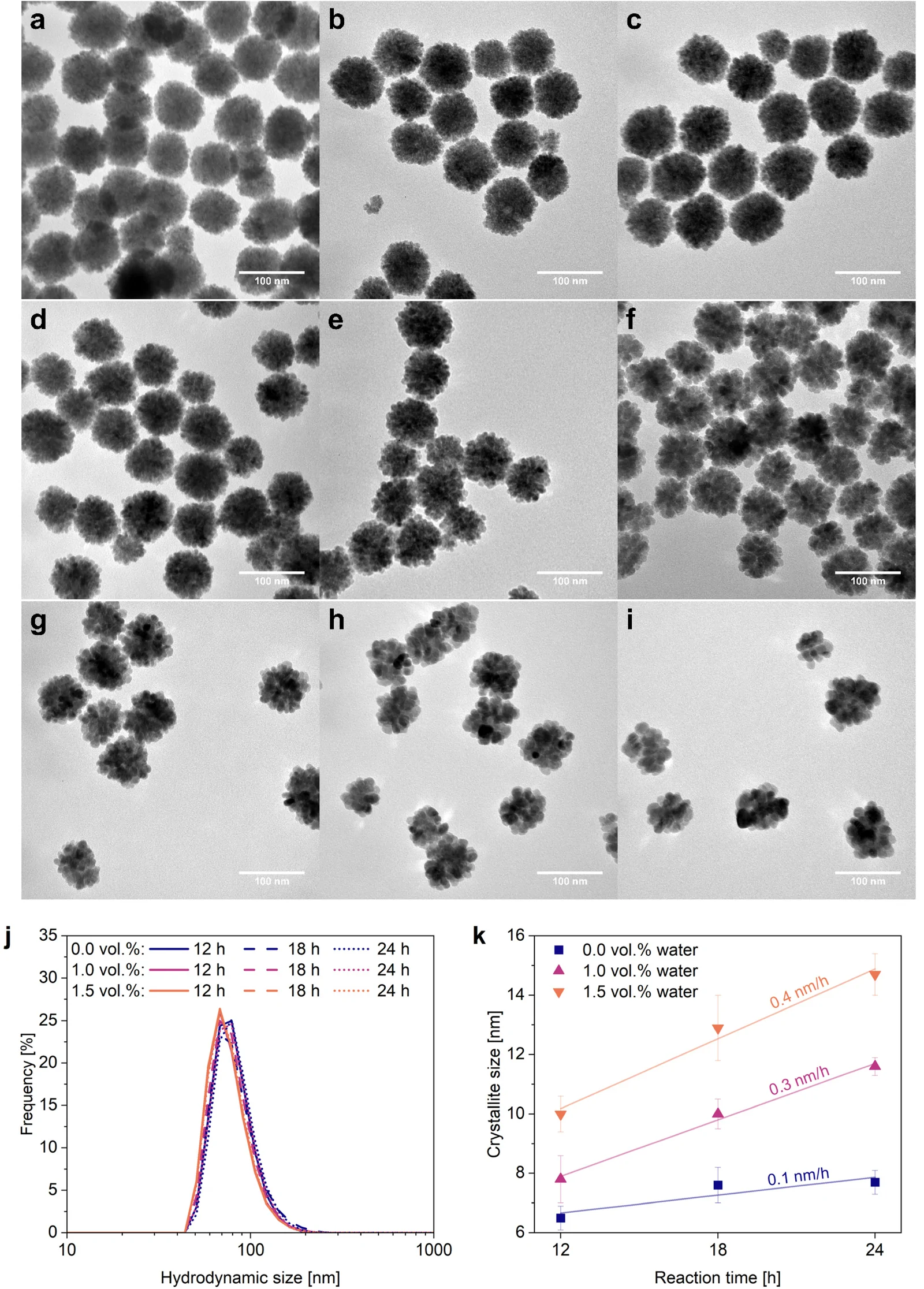
TEM images of the synthesized nanoclusters without additional water after 12 h (a), 18 h (b) and 24 h (c) along with the same time series with 1.0 vol% (d–f) and 1.5 vol% added water (g–i). DLS measurements indicate a constant nanocluster size throughout the reaction time (j), while the crystals grow further (Scherrer analysis-based values plotted) (k) indicating a ripening process.

This ripening process presents a second pathway besides sole water addition to control the crystallite size inside the nanoclusters. Unlike with water addition, the orientation of the crystallites remained coherent throughout the nanoclusters during the prolonged reaction time, thus enabling control over crystallite size and orientation (Fig. 6).

**Fig. 6:**
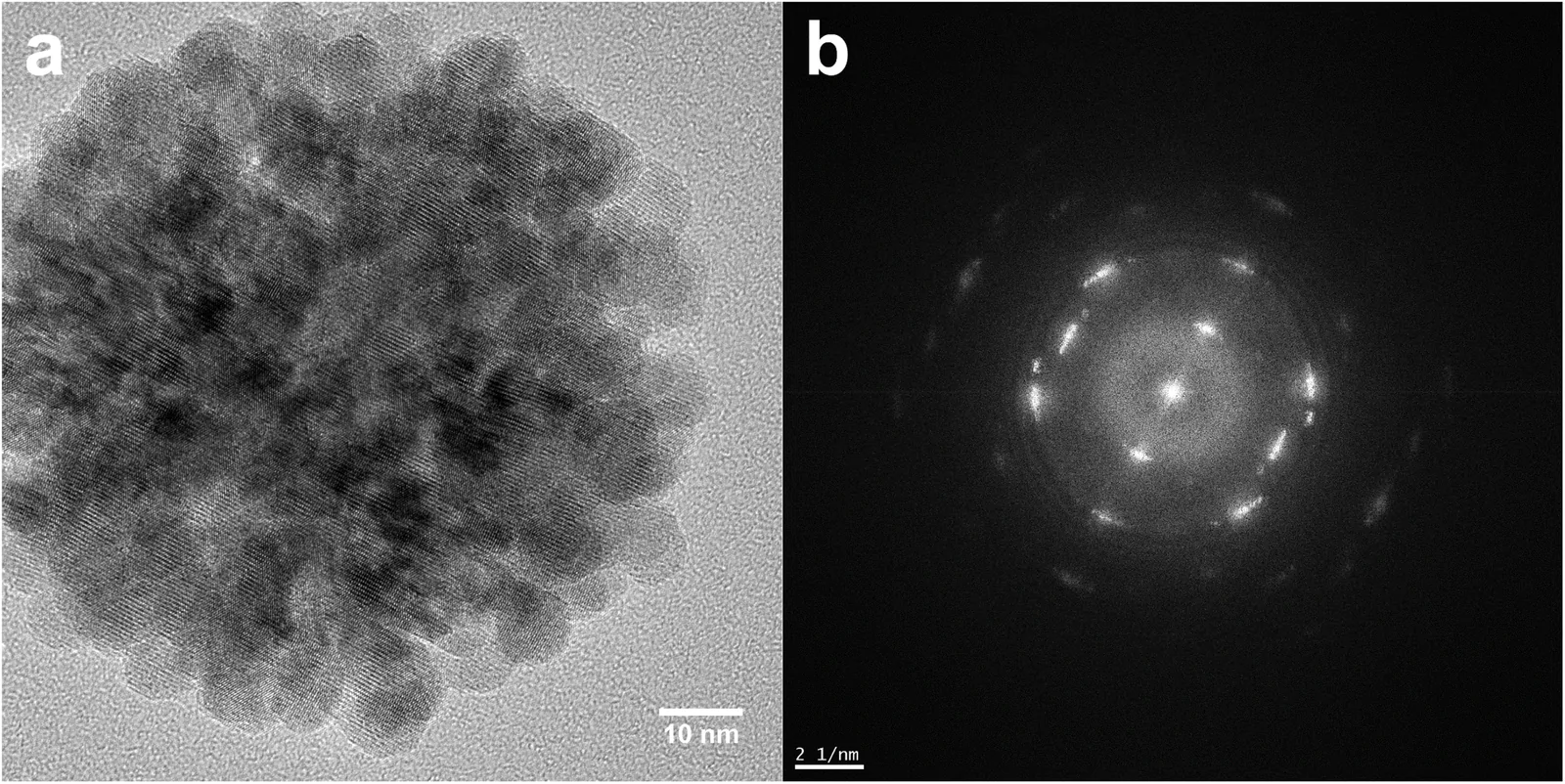
HRTEM image of the nanoclusters synthesized without additional water after 24 h reaction time (a) along with the FFT image (b). The whole image is used for the FFT.

## 4. Mechanistic implications

Based on the results described above, a reaction mechanism can be formulated as follows. As already discussed in the literature, the formation of monodisperse nanoclusters through aggregation of small iron oxide nanoparticles is the result of a delicate balance between attractive dispersion forces and repulsive electrostatic forces.^12–21^ During this process, the primary particles/crystallites aggregate through oriented attachment^23,43,45,87,88^ in a monomer-cluster growth mechanism. To achieve a tight packing of the crystallites with a coherent orientation resulting in the formation of well-defined spherical nanoclusters, reaction limited aggregation is necessary. This requires a strong enough electrostatic repulsion to balance the attractive dispersion forces that otherwise would lead to the formation of gel-like structures expected for diffusion limited growth. PEI induces these repulsive forces between the crystallites and in absence of PEI, the repulsion is decreased, leading to the creation of overall larger nanoclusters. Nevertheless, the repulsion is still within the range needed for the formation of well-defined clusters in the absence of PEI (Fig. 7a). When larger crystallites form upon water addition and the contribution of the attractive forces is enhanced, as these forces scale linearly with the radius for spherical particles.^89,90^ At some point, above a certain crystallite size, the balance between attractive and repulsive forces flips. In this case, the formation mechanism follows a diffusion limited pathway, resulting in open, less dense and irregular shaped aggregates where the crystallite orientation is more random (Fig. 7b). The effect of growing attractive forces facilitating the creation of less densely packed nanoparticle clusters has already been proposed in recent literature through theoretical simulations.^91^ Under the conditions studied here, the repulsive forces are high enough to allow the formation of spherical aggregates up to crystallite sizes of 11–13 nm in the presence of PEI (at 1.5 vol% water) and to about 9–10 nm in the absence of PEI (at 1.5 vol% water). It should be noted that the real cut-off values where the mechanism shifts from reaction to diffusion limited aggregation are probably smaller, since ripening has most likely already occurred after the initial aggregation step up to 12 h.

**Fig. 7:**
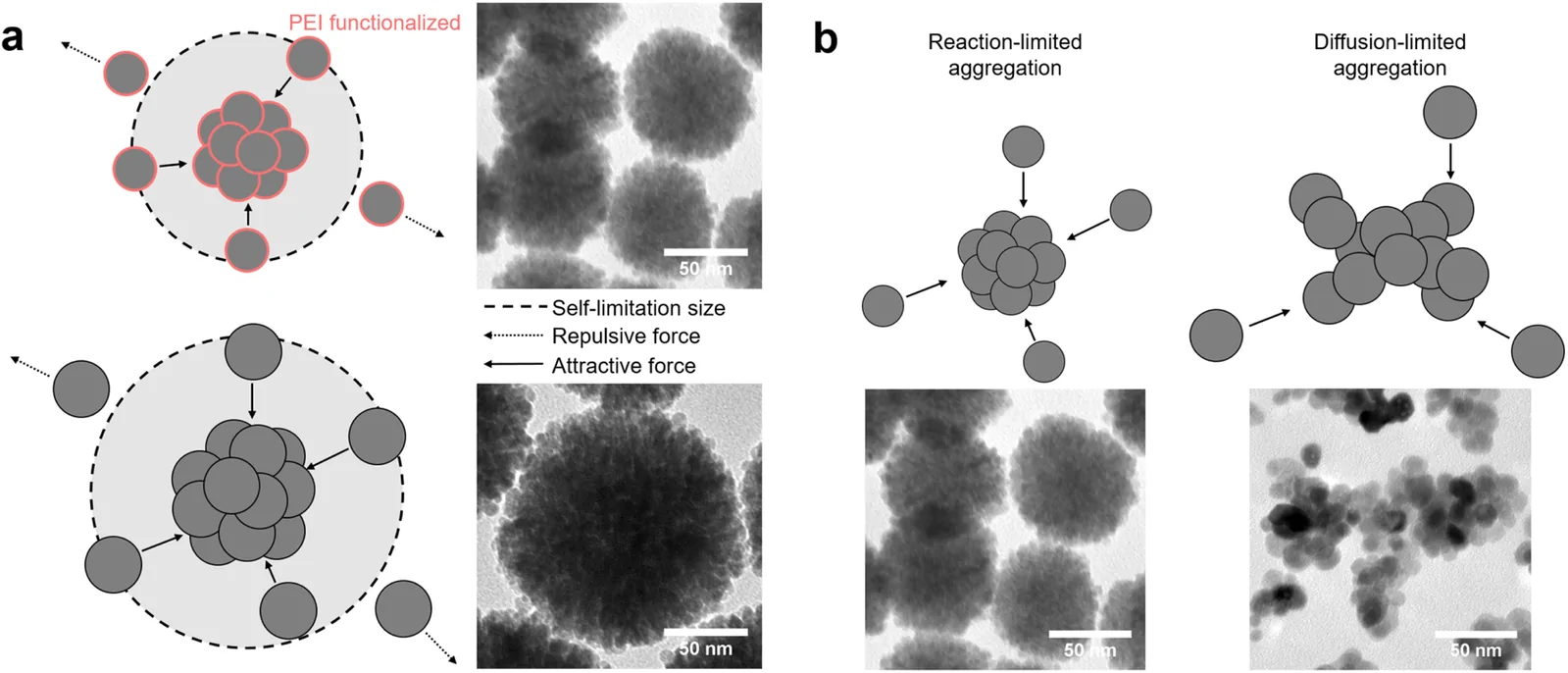
Illustration of the controlled aggregation from single crystallites to larger nanoclusters and the influence of long-range repulsive and short-range attractive forces that determine the self-limitation size. In the presence of PEI and small crystallites, the self-limitation occurs at lower sizes compared to the synthesis conducted in absence of PEI and with additional water, where larger crystallites allow the formation of larger nanoclusters (a). Small crystallites and thus low attractive forces lead to reaction limited compact aggregates, while large crystallites with increased attractive forces create rather diffusion limited open structures (b).

These findings are in agreement with other observations in literature, where for instance the choice of the solvent dictates whether the nanocluster formation follows a kinetically or thermodynamically driven reaction pathway, leading to a random or coherent crystallite orientation respectively.^43^ In this work, we focus especially on the crystallite size effect onto the aggregation mechanism. Others also argue that the surface ligands, especially their chemical nature, imposes an effect on the process of oriented attachment as well.^92^ However, we assume the surface of the synthesized nanoclusters is similarly covered with PEI in all samples independent of the water content as the zeta potentials measured in water were virtually identical for all samples, and also as the EDX maps show similar nitrogen distributions (Fig. S1b and S4).

According to our results, well-defined, spherical aggregates with larger crystallites and defined sizes could then be obtained, if such aggregates are allowed to undergo ripening during aging. During prolonged reaction times, the ripening kinetics increase with a higher water content in the reaction mixture. Meanwhile, the orientation of the crystallites does not change after the initial aggregation, opening up the possibility to control both the crystallite orientation as well as the size independently. We also note that the mechanism suggests that well-defined spherical aggregates with large crystallites should be obtainable under the condition that the electrostatic repulsion forces are higher than those prevailing under the conditions studied here. In that case, they could balance the increased attractive forces of large crystallites.

## 5. Conclusion

The synthesis and characterization of well-defined, spherical nanoclusters of superparamagnetic iron oxide nanoparticles is reported. The sizes of the clusters and the crystallites can be independently tuned within certain limits through rational tuning of the electrostatic repulsion and attractive dispersion forces during cluster formation. Further increases in the crystallite sizes can be achieved through ripening during aging, the kinetics of which is enhanced in the presence of water. If the primary particles/crystallites grow too large before aggregating into clusters, the size-dependent increase in attractive dispersion forces lead to the formation of more open, ill-defined nanoparticle aggregates, suggesting a transition from a reaction-controlled to a diffusion-controlled aggregation. The critical crystallite sizes depend on the magnitude of the repulsive electrostatic forces under the given conditions, where stronger electrostatic repulsion can tolerate larger crystallite sizes before the transition in aggregation mechanism occurs. However, the increase in attractive interactions with increasing primary particle size led to the formation of more open, surface fractal structures even in cases where well-defined spherical clusters formed. Thus, if dense clusters with larger crystallites are aimed for, the synthesis should involve ripening through aging. Through water content and reaction time, the crystallite size and orientation could be controlled simultaneously. Although not demonstrated here, we suggest that these mechanistic considerations are valid also for the formation of clusters of nanoparticles of other chemical compositions.

## Author contributions

Philipp Schwarz carried out the experiments and data analysis and wrote the manuscript. Heinz Amenitsch analyzed the SAXS data. Joachim Bansmann conducted XPS measurements and analysis. Johannes Biskupek made HRTEM images and EDX maps. Mika Lindén supervised the work and finalized the manuscript.

## Conflicts of interest

No conflicts of interest.

## Supplementary Material

RA-016-D6RA04862E-s001

## Data Availability

All data will be made available upon acceptance of the manuscript. Supplementary information (SI) is available. See DOI: https://doi.org/10.1039/d6ra04862e.
