# Supplementary material for: On the formation of superparamagnetic iron oxide nanoclusters
Source: RSC Adv. 2026 Oct 7;16(46):60706–17. doi: 10.1039/d6ra04862e (PMC13647222; doi:10.1039/d6ra04862e)
Supplement: Supplementary file 1 [file RA-016-D6RA04862E-s001.pdf]

Supplementary Information (SI) for RSC Advances.
This journal is © The Royal Society of Chemistry

Supplementary Information – On the formation of superparamagnetic iron oxide nanoclusters

Philipp Schwarz,[1] Heinz Amenitsch,[2] Joachim Bansmann,[3] Johannes Biskupek,[4] Mika Lindén[1,*]

[1] Institute of Inorganic Chemistry II, Ulm University, Albert-Einstein-Allee 11, 89081 Ulm, Germany

[2] Institute for Inorganic Chemistry, Graz University, Stremayrgasse 9/IV, 8010 Graz, Austria

[3] Institute of Physical Chemistry II, Ulm University, Albert-Einstein-Allee 47, 89081 Ulm, Germany

[4] Central Facility Electron Microscopy, Ulm University, Albert-Einstein-Allee 11, 89081 Ulm, Germany

* corresponding author: mlinden@uni-ulm.de

Nanocluster size and zeta potential

DLS shows monodisperse nanocluster structures. The zeta potential of all nanoclusters is around +30 mV, independent of the water content during synthesis due to highly cationic PEI on the nanocluster/crystallite surface. The observation that all nanoclusters possess a similar zeta potential is in agreement with the proposed mechanism that aggregation stops once the repulsive forces limit the addition of more crystallites to the cluster.

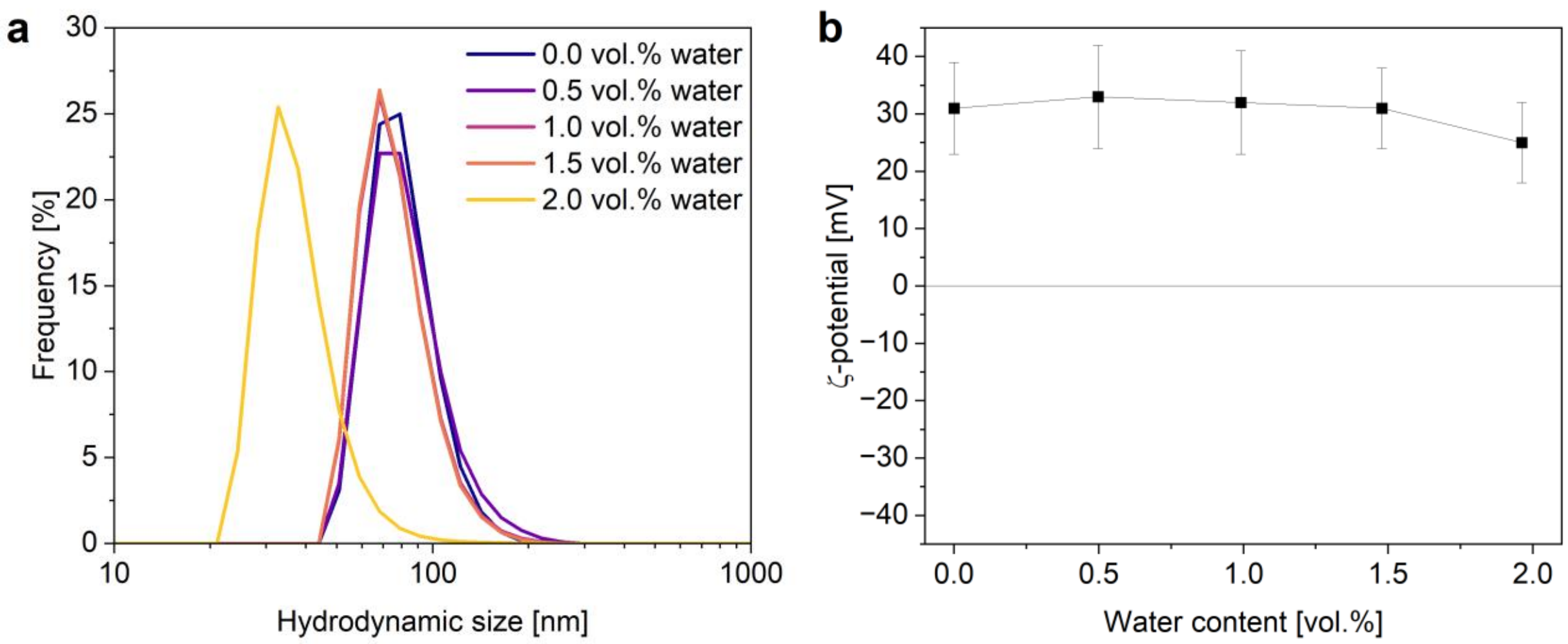

Fig. S1: DLS size analysis of nanoclusters synthesized with various amounts of additional water (a). The PEI functionalization is indicated by a highly positive zeta potential. Independent of the size and morphology, the resulting nanoclusters always show a zeta potential around values of +30 mV (b).

Iron oxide compositional analysis

XPS data analysis was carried out using the commercial XPS software CasaXPS (Version 2.3.23PR1.0). The spectra were referenced to the C-C / C-H signal from adventitious carbon at 284.8 eV. Background subtraction was carried out by using a Shirley background function. For the deconvolution of the different Fe oxides species, we followed the work of Biesinger et al.[1] and Hughes et al.[2] Since XRD data suggests either maghemite or magnetite as a possible material without any other detectable phases, other iron oxide species are ruled out in the deconvolution of XPS signals. Magnetite tends to oxidize towards maghemite on the outer shell. Small crystallites like in the sample prepared without any additional water (0.0 vol.%) thus show a larger maghemite share, while large crystallites formed with 1.5 vol.% water possess a lower surface-to-volume ratio and are thus more magnetite-rich.

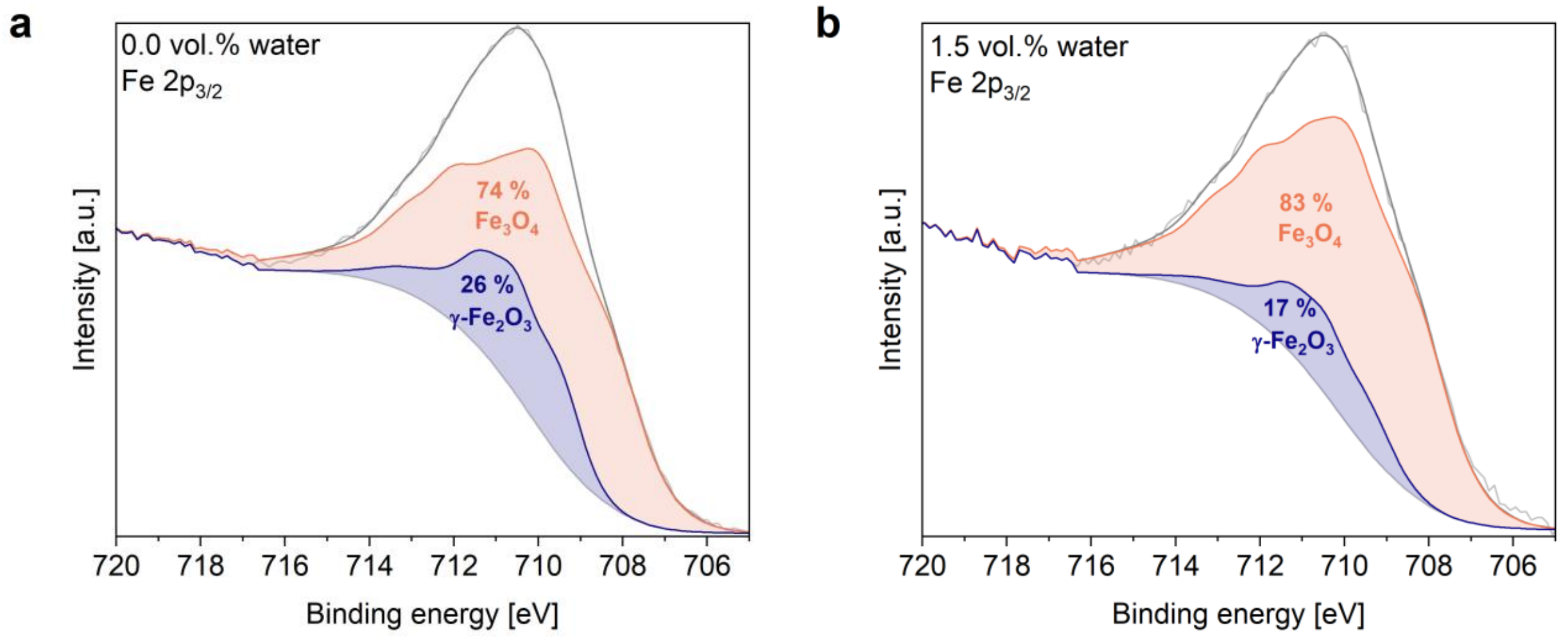

Fig. S2: XPS spectra of the iron oxide nanoclusters synthesized without additional water (a) and with 1.5 vol.% water in the reaction mixture (b) along with the share of magnetite and maghemite according to XPS.

Raman spectra were analyzed based on literature known peak positions of maghemite and magnetite.[3–6] Based on the spectra recorded with a 532 nm laser, the material can be classified as mainly maghemite according to a strong peak at 713 – 715 cm^{-1}. With a 633 nm laser wavelength, also magnetite-specific peaks at 670 – 680 cm^{-1} appear. These findings might be explained through a rather surface-sensitive analysis at 532 nm and a more bulk-sensitive analysis at 633 nm.[7] Thus, the surface of the material is maghemite-rich while the core is more magnetite-rich.

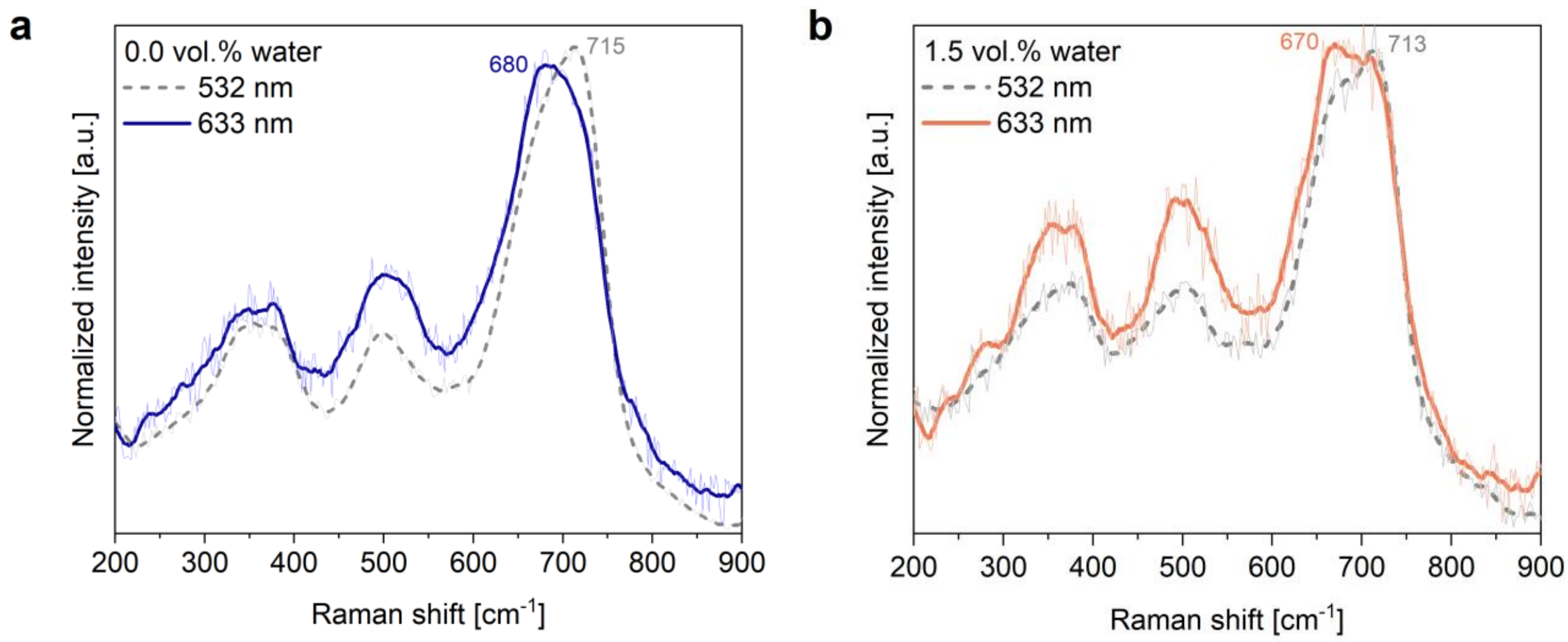

Fig. S3: Raman spectra of the nanoclusters synthesized with 0.0 vol.% (a) and 1.5 vol.% water (b) indicates a rather maghemite-rich surface (532 nm laser) and a magnetite-rich core (633 nm laser) in both cases.

EDX elemental mapping in TEM showed a uniform Fe and O distribution. Nitrogen is also located within the cluster structures, indicating the presence of PEI as a surface polymer around the crystallites. The ratio of Fe to O is around 3 to 4, further suggesting the presence of magnetite. There are also traces of Mg and Si, probably induced through the addition of water during the synthesis.

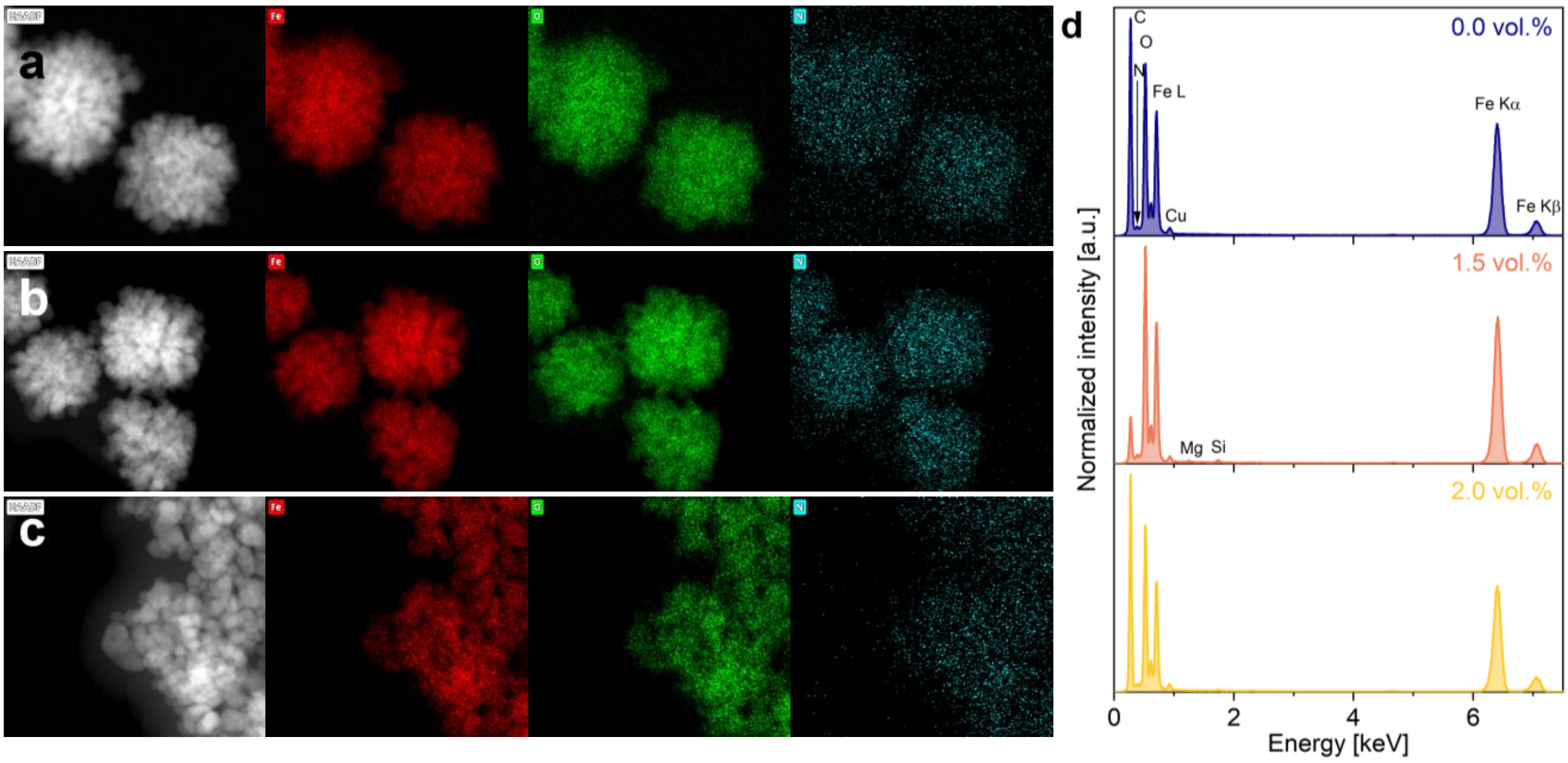

Fig. S4: Elemental mapping of nanoclusters synthesized without (a), 1.5 vol.% (b) and 2.0 vol.% water (c). Iron (red), oxygen (green) and nitrogen (blue) are uniformly distributed, suggesting both the presence of magnetite as well as PEI. The EDX spectra shows the abundance of each element (d).

Magnetic properties

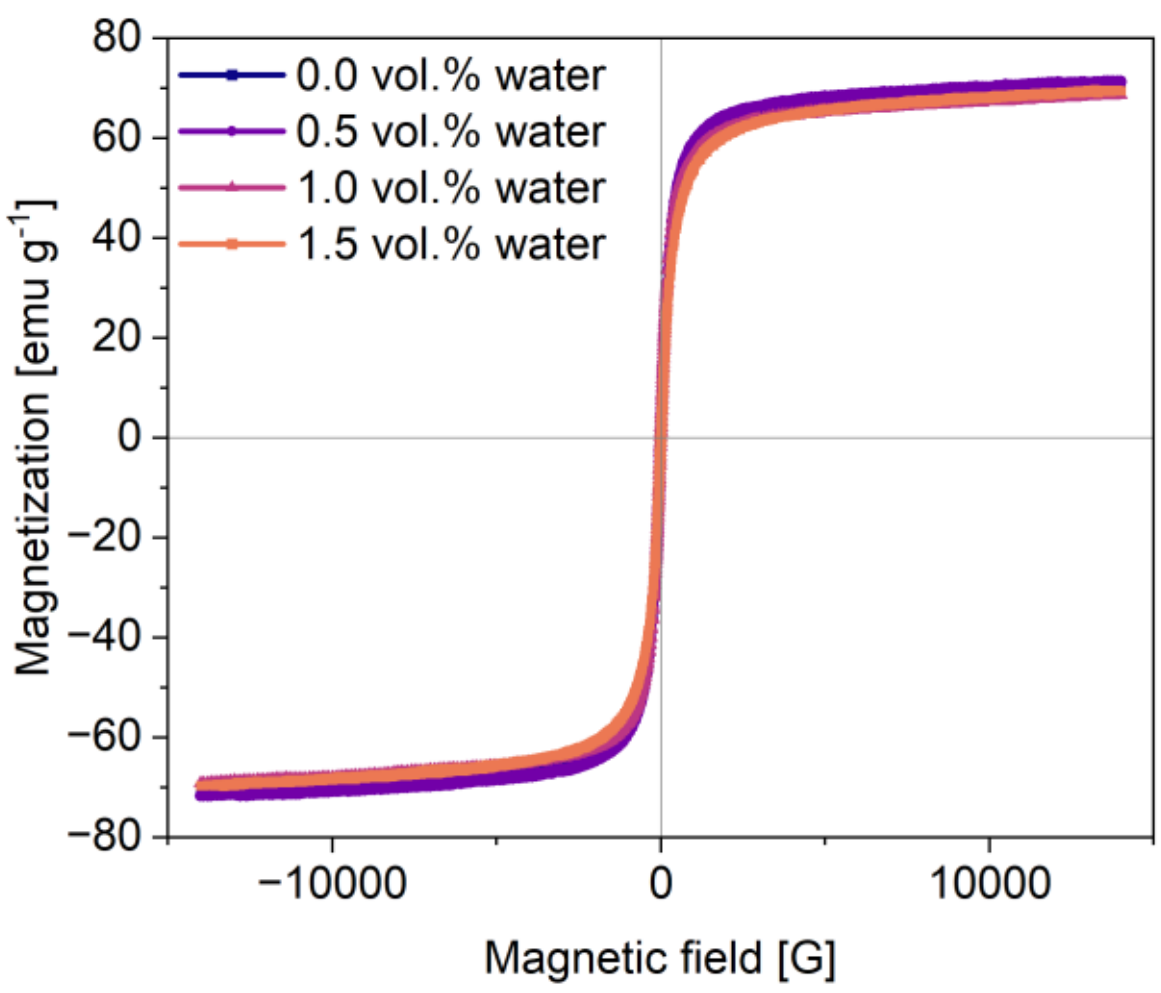

Fig. S5: VSM measurements of the different nanoclusters synthesized with various water contents. Normalized to the pure iron oxide mass based on thermogravimetry.

Crystallite size and strain analysis

With increased water content, a narrowing of the reflections can be observed. The width of the reflections is influenced by the size as well as strain. A simple size calculation based on the Scherrer equation neglects the latter one. There are several approaches to isolate size and strain contributions. The Williamson-Hall plot is the most well-known size-strain-analysis technique. If the size effect is assumed to dominate, a Cauchy (lorentzian) peak profile is assumed and the linear Williamson-Hall plot is used for analysis (Fig. S6a):

$$\frac{\beta_{hkl}\cos(\theta)}{\lambda} = \frac{K}{D} + \frac{4\varepsilon\sin(\theta)}{\lambda} \quad (1)$$

When the strain dominates, a Gaussian peak profile fit is used along with the quadratic Williamson-Hall equation (Fig. S6b):

$$\left(\frac{\beta_{hkl}\cos(\theta)}{\lambda}\right)^2 = \left(\frac{K}{D}\right)^2 + \left(\frac{4\varepsilon\sin(\theta)}{\lambda}\right)^2 \quad (2)$$

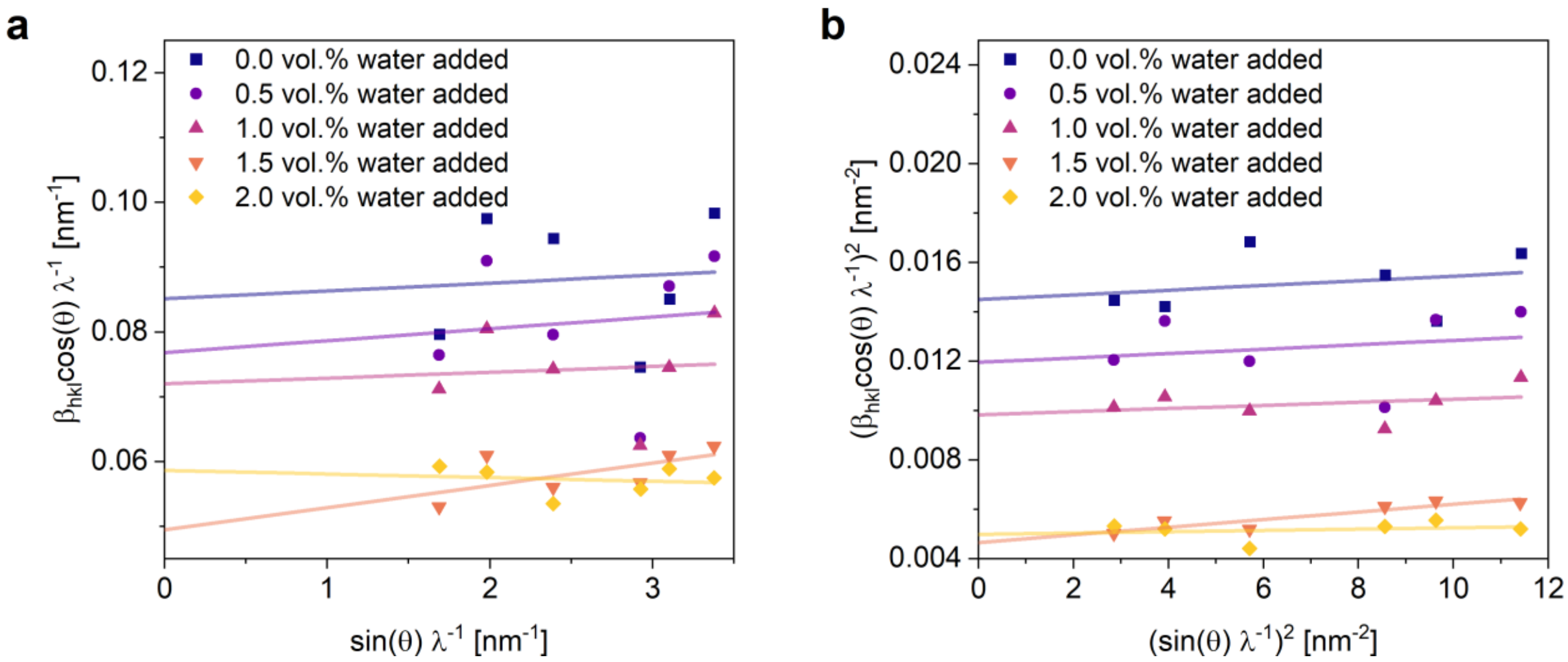

Fig. S6: Williamson-Hall plot based on the linear equation and a Cauchy (lorentzian) peak profile fit (a) along with the quadratic form and a Gaussian profile fit (b).

Both approaches based on the Williamson-Hall analysis yielded poor results in terms of mathematical accuracy (R^2) as well as resulting in physically inconsistent values. On the other hand, the so-called size-strain plot, derived by the dimensionless Halder-Wagner equation and used in several literature sources, represents another way to determine size and strain contributions to the reflection FWHM in XRD.[8–10] It should be mentioned that many publications rely on the dimensionally incorrect version of the size-strain equation.[10] In comparison to the Williamson-Hall analysis, this approach relies on a Pseudo-Voigt profile fit and puts more weight on reflections at low angles. Next to that, the crystallite size is now determined by the slope and not by the y-intercept.

$$\left(\frac{d_{hkl}\beta_{hkl}\cos(\theta)}{\lambda}\right)^2 = \frac{K}{D}\left(\frac{d_{hkl}^2\beta_{hkl}\cos(\theta)}{\lambda}\right) + \frac{\varepsilon^2}{4} \quad (3)$$

This size-strain analysis gave better statistics than did the Williamson-Hall analysis.

Table S1: Comparison of the derived parameters using the Williamson-Hall plot and the size-strain analysis.

Water added [vol.%]	Williamson-Hall plot (linear, Cauchy)			Williamson-Hall plot (quadratic, Gaussian)			Size-strain-analysis (Pseudo-voigt)		
	Size [nm]	Strain [%]	R^2	Size [nm]	Strain [%]	R^2	Size [nm]	Strain [%]	R^2
0.0	11	0.005	0.0066	7.5	0.04	0.0640	8.2	1.2	0.9890
0.5	12	0.007	0.0131	8	0.04	0.0416	9.1	1.0	0.9966
1.0	12	0.003	0.0068	9.1	0.03	0.0956	10.6	1.2	0.9997
1.5	18	0.013	0.3972	13.2	0.05	0.8324	13.3	0.6	0.9991
2.0	15	-0.002	0.0277	13	0.02	0.0538	14.1	0.4	0.9915

The size-strain plot shows that the broadening of the reflection is partially caused by a high strain, especially for small crystallite sizes. Larger crystallites obtained at higher water contents then show lower strain values. Thus, the Scherrer method systematically underestimates the crystallite size especially for smaller crystallites.

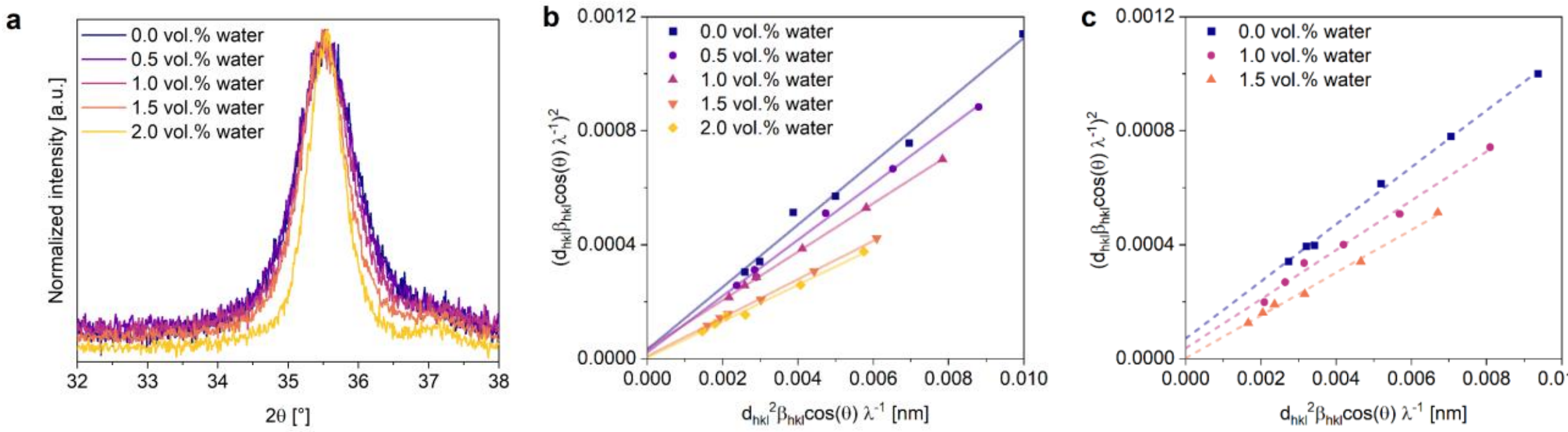

Fig. S7: A narrowing of the 311 reflection can be observed with increased water addition during synthesis (a). Based on a size-strain plot, the size and strain effect on the reflection width can be isolated (b). If PEI is absent during the reaction, a similar trend in crystallite growth can be observed with additional water (c).

With increasing reaction time, a decreasing slope can be seen in the size-strain plots, indicating a crystallite growth. If the crystallite size is then plotted against the reaction time, an accelerated ripening with additional water can be seen. Both the size-strain plot as well as the Scherrer analysis thus suggest a ripening process inside the clusters throughout time. Without additional water a size increase of 0.1 nm/h can be determined. With increasing water addition, this growth accelerates to approximately 0.3 nm/h at 1.0 vol.% and 0.5 nm/h at 1.5 vol.% water.

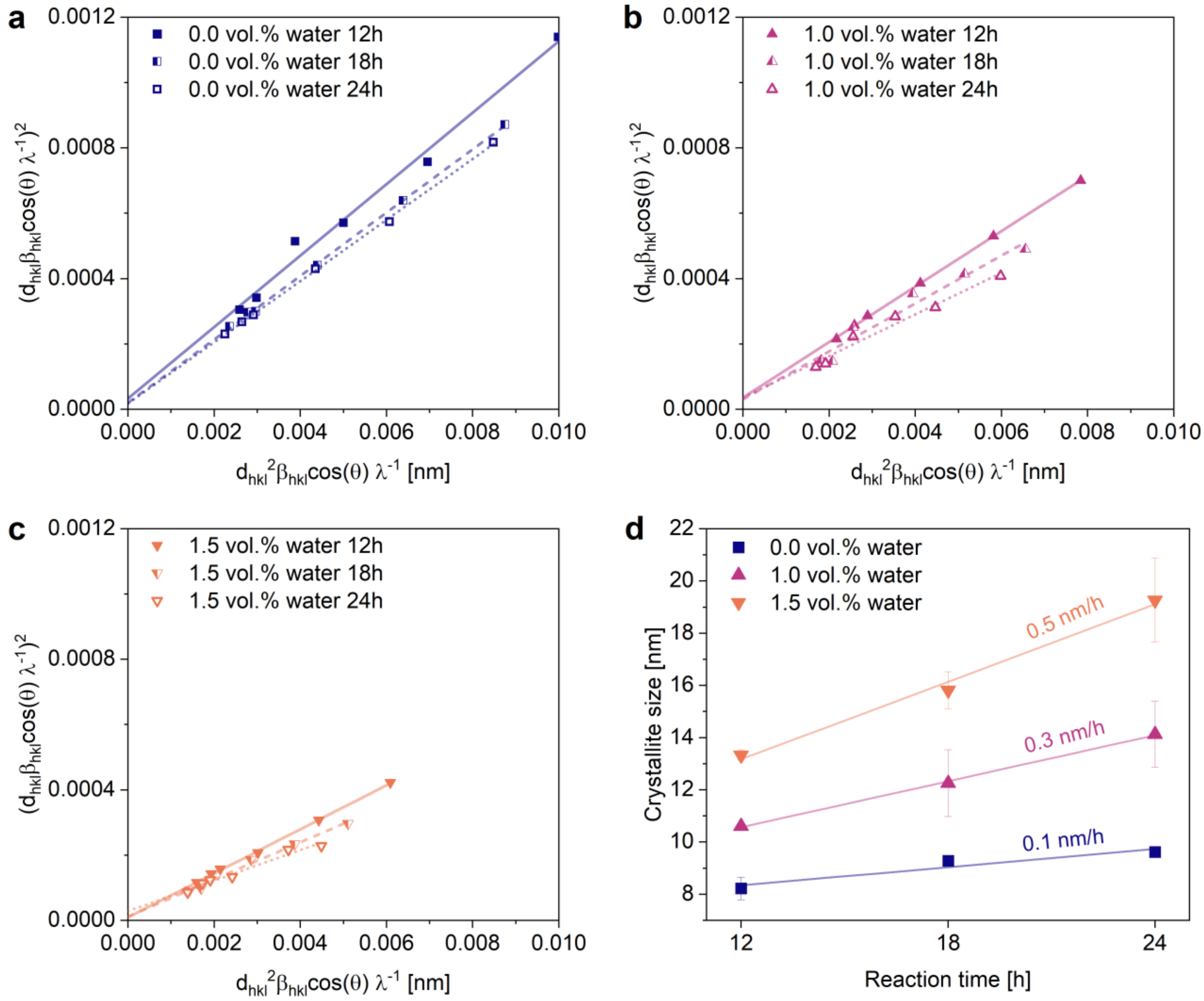

Fig. S8: Size-strain plots of the samples with various additional water contents of 0.0 vol.% (a), 1.0 vol.% (b) and 1.5 vol.% (c) after different reaction times indicate a crystallite growth through prolonged reaction times. If the crystallite size is plotted against the reaction time, an accelerated crystallite growth can be detected based on the sizes derived by the size-strain plot (d).

HRTEM and crystallite orientation

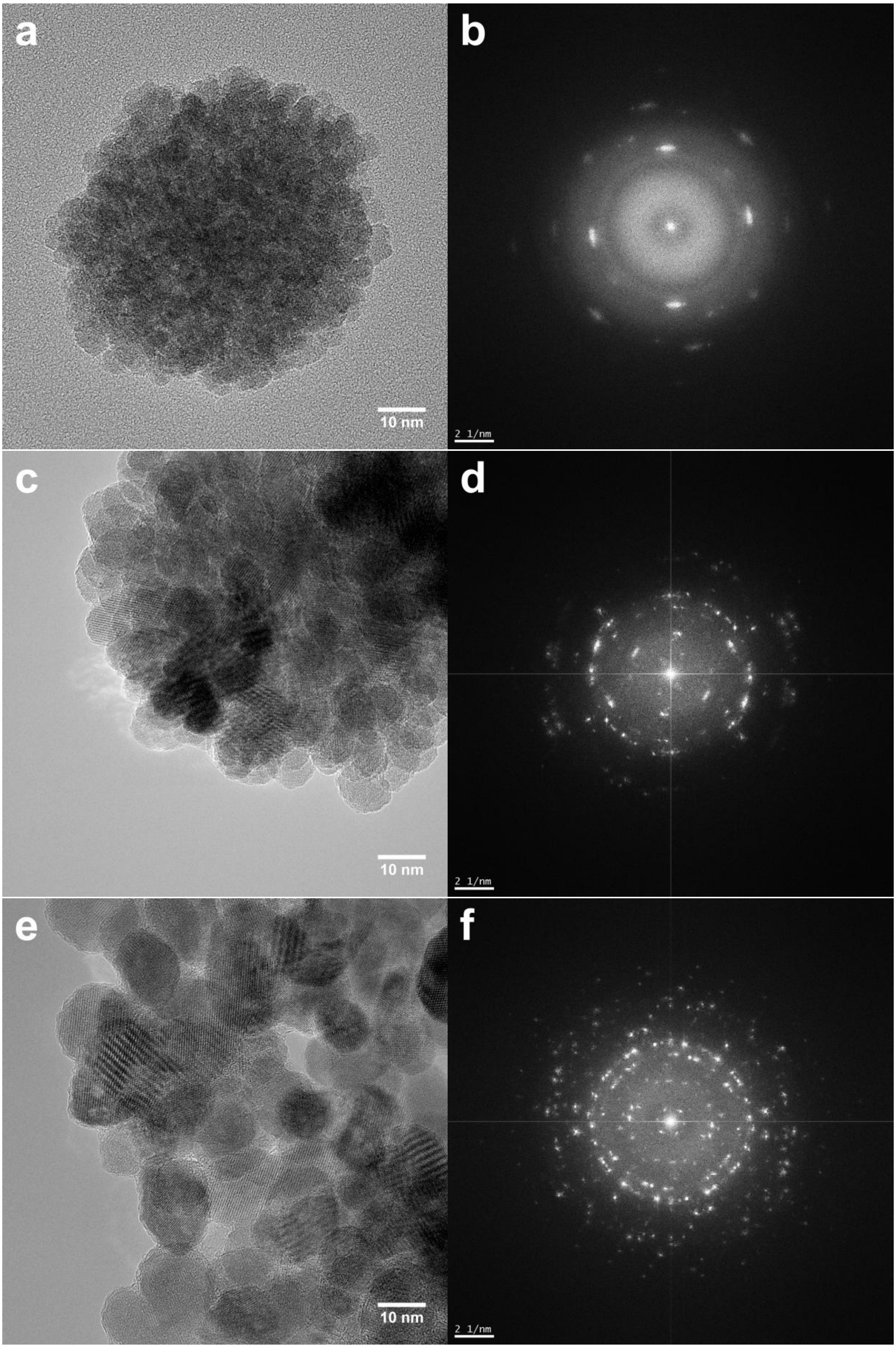

Fig. S9: Additional HRTEM images of the samples with 0.0 vol.% (a), 1.5 vol.% (b) and 2.0 vol.% (c) additional water along with the corresponding FFT images of the shown nanoclusters (d – f). The FFT images are based on the whole image.

HRTEM crystallite size analysis

HRTEM allows to graphically analyze the crystallites. 50 visible crystallites were measured for each sample to approximate a mean size value along with a standard deviation value. The width of the size distribution increases with growing crystallites.

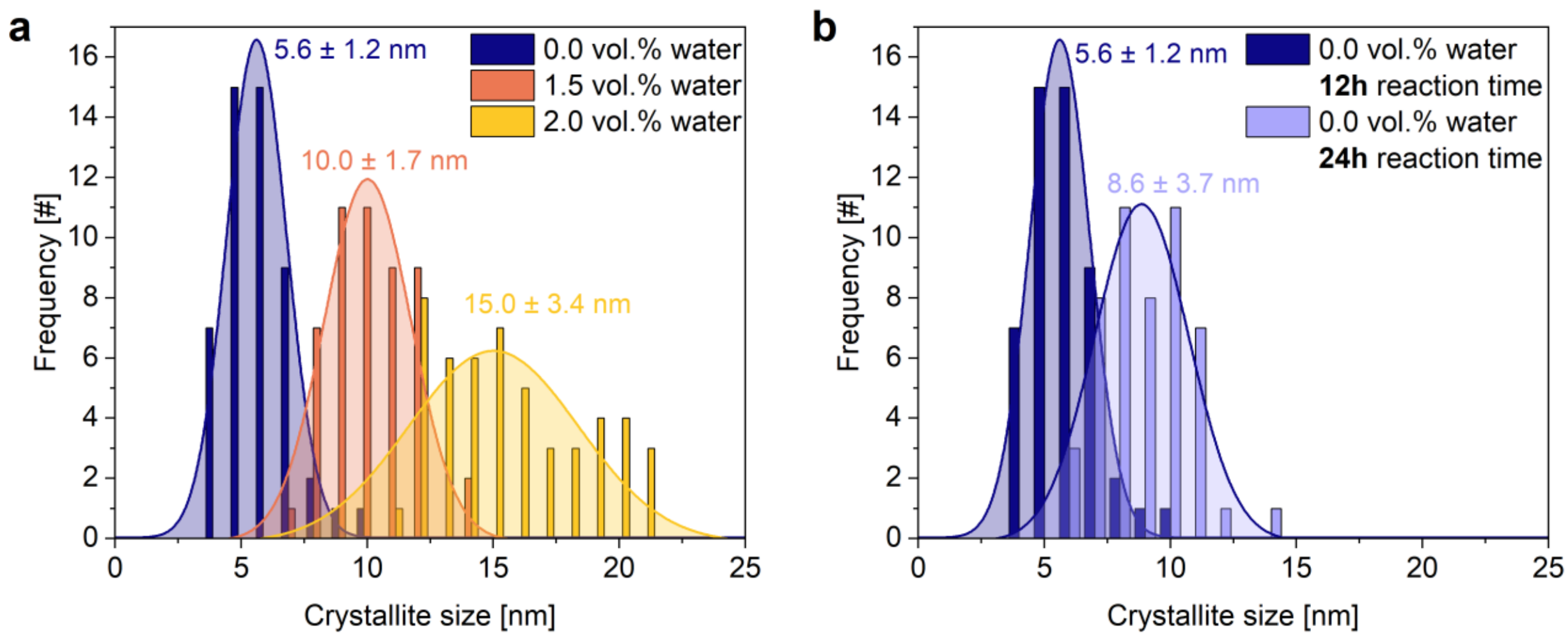

Fig. S10: Crystallite sizes based on HRTEM of the 0.0 vol.%, 1.5 vol.% and 2.0 vol.% samples after 12 h reaction time (a). With prolonged reaction times, an increase in crystallite size can be observed (b). The mean size values of each sample obtained by a Gauss fit are shown together with the standard deviation.

SAXS analysis

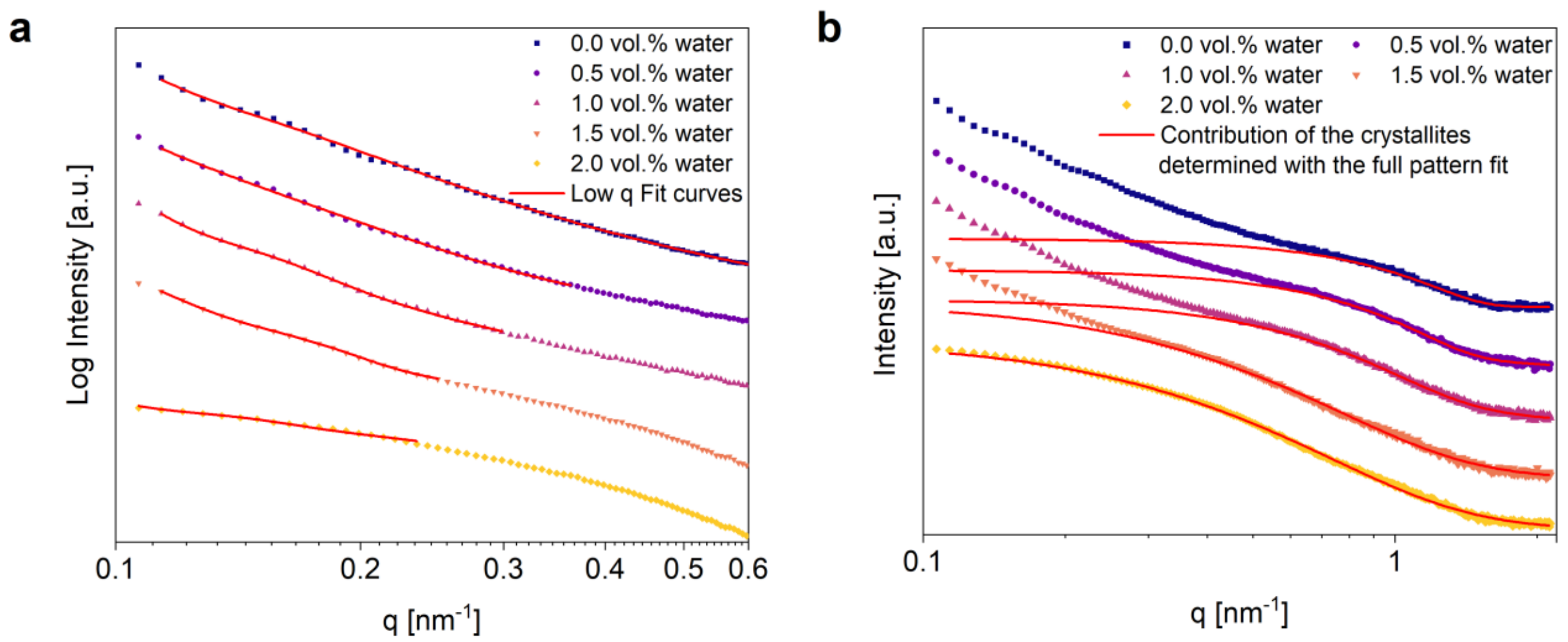

Fig. S11: SAXS curves along with the low q fit curves used to analyze the aggregate structures (a) along with the contribution of the crystallites determined with the full pattern fit (b).

References

1. M. C. Biesinger, B. P. Payne, A. P. Grosvenor, L. W. Lau, A. R. Gerson, R. S. Smart, *Appl. Surf. Sci.,* 2011, **257**(7), 2717–30, DOI: 10.1016/j.apsusc.2010.10.051.

2. A. E. Hughes, C. D. Easton, T. R. Gengenbach, M. C. Biesinger, M. Laleh, *J. Vac. Sci. Technol., A,* 2024, **42**(5), DOI: 10.1116/6.0003804.

3. S. P. Schwaminger, D. Bauer, P. Fraga-García, F. E. Wagner, S. Berensmeier, *CrystEngComm,* 2017, **19**(2), 246–55, DOI: 10.1039/c6ce02421a.

4. M. Sainz-Menchón, I. González de Arrieta, T. Echániz, K. Nader, M. Insausti, A. Canizarès et al., *Phys. Chem. Chem. Phys.,* 2025, **27**(16), 8498–509, DOI: 10.1039/d5cp00503e.

5. S. P. Schwaminger, P. Fraga-García, F. Selbach, F. G. Hein, E. C. Fuß, R. Surya et al., *Adsorption,* 2017, **23**(2-3), 281–92, DOI: 10.1007/s10450-016-9849-y.

6. O. N. Shebanova and P. Lazor, *J. Solid State Chem.,* 2003, **174**(2), 424–30, DOI: 10.1016/S0022-4596(03)00294-9.

7. R. Otero-Lorenzo, E. Fantechi, C. Sangregorio, V. Salgueiriño, *Chemistry,* 2016, **22**(19), 6666–75, DOI: 10.1002/chem.201505049.

8. S. A. Hassanzadeh-Tabrizi, *J. Alloys Compd.,* 2023, **968,** 171914, DOI: 10.1016/j.jallcom.2023.171914.

9. D. H. Manh, T. T. Ngoc Nha, T. Le Hong Phong, P. H. Nam, T. D. Thanh, P. T. Phong, *RSC Adv.,* 2023, **13**(36), 25007–17, DOI: 10.1039/d3ra04018f.

10. A. Pal, *J. Alloys Compd.,* 2025, **1048,** 185324, DOI: 10.1016/j.jallcom.2025.185324.
